# Supplementary material for: Long-term psychological distress trajectories and the COVID-19 pandemic in three British birth cohorts: A multi-cohort study
Source: PLoS Med. 2023 Apr 4;20(4):e1004145. doi: 10.1371/journal.pmed.1004145 (PMC10072377; doi:10.1371/journal.pmed.1004145)
Supplement: S1 Supporting Information — Appendix A. Information on the psychological distress measures used in the study. Appendix B. Harmonised items across psychological distress measures in NSHD. Appendix C. Additional psychological distress operationalisations used in sensitivity checks. Figure C1. Proportion of NSHD participants endorsing each GHQ-12 item in the COVID-19 Survey waves. Table C1. Weighted prevalence estimates (and 95% CIs) of psychological distress “caseness” across COVID-19 Survey waves by operationalisation. Appendix D. Measurement invariance testing. Table D1. Malaise inventory measurement invariance testing results. Table D2. GAD-2 and PHQ-2 measurement invariance testing results. Appendix E. STROBE checklist for cohort studies. Appendix F. Number of missing observations by cohort and wave. Appendix G. Results from multilevel growth curve models with cross-cohort factor scores as outcome (linear models). Table G1. Marginal mean levels predicted from the multilevel growth curve models with cross-cohort factor scores as outcome (linear models). Figure G1. Marginal mean cross-cohort psychological distress factor scores over time (year) by birth sex. Appendix H. Results from multilevel growth curve models with cross-cohort factor scores as outcome (linear models), sensitivity checks with extended non-response weights. Table H1. Model coefficients from the multilevel growth curve models with cross-cohort factor scores as outcome (linear models), sensitivity checks with extended non-response weights. Table H2. Marginal mean levels predicted from the multilevel growth curve models with cross-cohort factor scores as outcome (linear models), sensitivity checks with extended non-response weights. Appendix I. Results from multilevel growth curve models with number of symptoms as outcome (Poisson models). Table I1. Model coefficients from the multilevel growth curve models with number of symptoms as outcome (Poisson models). Table I2. Marginal mean levels predicted from the multilevel growth c [file pmed.1004145.s001.pdf]

Long-term psychological distress trajectories and the COVID-19 pandemic in three British birth cohorts: a multi-cohort study

S1 Supplementary Material

Table of Contents

Appendix A. Information on the psychological distress measures used in the study 3

Appendix B. Harmonised items across psychological distress measures in NSHD 4

Appendix C. Additional psychological distress operationalisations used in sensitivity checks 5

Figure C1. Proportion of NSHD participants endorsing each GHQ-12 item in the COVID-19 Survey waves 6

Table C1. Weighted prevalence estimates (and 95% CIs) of psychological distress 'caseness' across COVID-19 Survey waves by operationalisation 6

Appendix D. Measurement invariance testing... 7

Table D1. Malaise inventory measurement invariance testing results 8

Table D2. GAD-2 and PHQ-2 measurement invariance testing results 8

Appendix E. STROBE checklist for cohort studies... 9

Appendix F. Number of missing observations by cohort and wave 11

Appendix G. Results from multilevel growth curve models with cross-cohort factor scores as outcome (linear models).. 12

Table G1. Marginal mean levels predicted from the multilevel growth curve models with cross-cohort factor scores as outcome (linear models) 12

Figure G1. Marginal mean cross-cohort psychological distress factor scores over time (year) by birth sex.. 13

Appendix H. Results from multilevel growth curve models with cross-cohort factor scores as outcome (linear models), sensitivity checks with extended non-response weights... 14

Table H1. Model coefficients from the multilevel growth curve models with cross-cohort factor scores as outcome (linear models), sensitivity checks with extended non-response weights... 14

Table H2. Marginal mean levels predicted from the multilevel growth curve models with cross-cohort factor scores as outcome (linear models), sensitivity checks with extended non-response weights 15

Appendix I. Results from multilevel growth curve models with number of symptoms as outcome (Poisson models) 16

Table I1. Model coefficients from the multilevel growth curve models with number of symptoms as outcome (Poisson models) 16

Table I2. Marginal mean levels predicted from the multilevel growth curve models with number of symptoms as outcome (Poisson models) 17

Figure I1. Marginal mean number of psychological distress symptoms over time (year and age) 18

Appendix J. Results from multilevel growth curve models with caseness as outcome (logistic models) 19

Table J1. Model coefficients from the multilevel growth curve models with caseness as outcome (logistic models).. 19

Table J2. Marginal mean levels predicted from the multilevel growth curve models with caseness as outcome (logistic models)... 20

Figure J1. Marginal predicted mean probability of psychological distress over time (year and age).. 21

Appendix K. Results from multilevel growth curve models with factor scores as outcome (linear models), 7 harmonised psychological distress indicators in NSHD 22

Table K1. Model coefficients from the multilevel growth curve models with factor scores as outcome (linear models), 7 harmonised indicators in NSHD 22

Table K2. Marginal mean levels predicted from the multilevel growth curve models with factor scores as outcome (linear models), 7 harmonised indicators in NSHD 22

Figure K1. Marginal mean psychological distress factor scores over time (year), 7 harmonised psychological distress indicators in NSHD 23

Additional references 24

Appendix A. Information on the psychological distress measures used in the study

Cohort	Measure	Details	'Caseness' threshold	Psychometric and clinical properties
**NSHD**	Present State Examination (PSE)	Nurse-administered clinical examination assessing the frequency and severity of a range of psychiatric symptoms in the preceding month. Used at age 36.	5 or higher on the Index of Definition [1].	Tested in general population: high agreement with clinical diagnosis of common mental health disorders (~90%); high concurrent validity with other measures of psychological distress [1].
	Psychiatric Symptoms Frequency (PSF)	Nurse-administered questionnaire (18 items) assessing symptoms of anxiety and depression during the preceding year. 5-point scale from 0 ("Never in the last year") to 5 ("Every day in the last year"). Used at age 43.	23 or higher on the summed up score [2].	Tested in general health care/general population: Cronbach's alpha = 0.88; all items identified a common factor; AUC against reports of contact with doctor/use of prescribed medication for 'nervous or emotional trouble or depression' = 0.84-0.86 [2].
	General Health Questionnaire, 28-item version (GHQ-28)	Self-administered questionnaire (28 items) assessing symptoms of anxiety in depression in the preceding 4 weeks. 4-point scale recoded into binary values [3]. Used at ages 50-69.	6 or higher score [3].	Tested in general health care (adults): Cronbach's alpha = 0.82-0.86; AUC against diagnosed psychiatric morbidity = 0.88 [3].
	General Health Questionnaire, 12-item version (GHQ-12)	Self-administered questionnaire (12 items) assessing whether symptoms of anxiety and depression were 'recently' experienced. 4-point scale recoded into binary values [3]. Used at ages 74-75.	2 or higher score [3].	Tested in general health care (adults): AUC against diagnosed psychiatric morbidity = 0.88 [3].
**NCDS and BCS70**	Malaise Inventory	Self-administered questionnaire (9 items) assessing whether distressful experiences are 'generally' experienced. Used at all ages from 23 (NCDS) and 26 (BCS70) onwards.	4 or higher on the summed-up score	Tested in general population, the full 24-item version showed an AUC against self-reported diagnosed psychiatric morbidity = 0.77-0.79 [4]. The 9-item version correlates highly (r=0.91-0.92) with the 24-item version [5].
**NSHD, NCDS, and BCS70**	Generalized Anxiety Disorder, 2-item version (GAD-2)	Self-administered questionnaires (2 items) assessing two core anxiety and depressive symptoms, respectively, over the last 2 weeks. 4-point scale from 1 ("Not at all") to 4 ("Nearly every day"). Used at all three COVID-19 Survey waves.	Not applicable. These items are used in the factor score approach with the three cohorts as indicators of the latent level of psychological distress due to their presence in all three cohorts at the same three time points ('anchor' items). Therefore, no 'caseness' thresholds are used.	Tested in general health care (adults): AUC against major depressive disorder diagnostic = 0.93, against any depressive disorder = 0.90; correlation with functional impairment due to mental health issues (r=0.70) [6].
	Patient Health Questionnaire, 2-item version (PHQ-2)			Tested in general health care (adults): AUC against generalised anxiety disorder = 0.91, against any anxiety disorder = 0.85 [7].

Note. Adapted from Gondek at al. [8]

Appendix B. Harmonised items across psychological distress measures in NSHD

Symptom	Present State Examination (PSE)	Psychiatric Symptom Frequency (PSF)	General Health Questionnaire, 28-item version (GHQ-28)	General Health Questionnaire, 12-item version (GHQ-12)
Low Mood	Do you keep reasonably cheerful or have you been very depressed or low spirited recently? (rate depressed mood)	Over the last year have you been in low spirits or felt miserable?	Have you recently been able to enjoy your normal day-to-day activities?	Have you recently been feeling unhappy or depressed? *
Fatigue	Have you been exhausted and worn out during the day or evening even when you haven't been working very hard? (rate tiredness/exhaustion)	Over the last year have there been days when you tired out very easily?	Have you recently been feeling in need of a good tonic?	
Tension	Do you often feel on edge, keyed up, mentally tense or strained? (rate nervous tension)	Over the last year have you felt on edge, keyed up or mentally tense?	Have you recently felt constantly under strain?	Have you recently felt constantly under strain?
Panic	Have you had times when you felt shaky or you heart pounded or you felt sweaty and you simply had to do something about it?	Over the last year have you been in situations when you felt shaky or sweaty or your heart pounded or you could not get your breath?	Have you recently been getting scared or panicky for no good reason?	
Hopelessness	How do you see the future? (rate hopelessness)	Over the last year have you had the feeling that the future does not hold much for you?	Have you recently felt that life is entirely hopeless?	
Health anxiety	Do you tend to worry over your physical health? (rate hypochondriasis)	Over the last year have you been frightened or worried about becoming ill or about dying?	Have you recently felt that you are ill?	
Sleep problems	Have you had any trouble getting off to sleep in the last month? (rate delayed sleep)	Over the last year have you had trouble getting off to sleep?	Have you recently lost much sleep over worry?	Have you recently lost much sleep over worry?
Response options	Symptom not present/ Symptom definitely present during past month, but of moderate clinical intensity/ Intense form of symptom present for more than 50% of past month	Never/ Occasionally/ Sometimes/ Quite often/ Very often/ Always	Not at all/No more than usual/ Rather more than usual/ Much more than usual	Not at all/No more than usual/ Rather more than usual/ Much more than usual

Note. Adapted from Gondek et al. [8] and McElroy et al. [9] Additional details on the harmonisation procedure are available in McElroy et al. [9] * Although the question used to represent the "low mood" symptom was present in both the GHQ-28 and GHQ-12, this item was deemed inadequate to adequately reflect the person's mood at the first COVID-19 Survey wave, seemingly being more reflective of the current enforced restrictions legally limiting the ability of the person to perform their normal day-to-day activities. More information on this is available in the Appendix S3 in this same document.

Appendix C. Additional psychological distress operationalisations used in sensitivity checks

As sensitivity checks, we used alternative psychological distress operationalisations, in addition to the main operationalisation as a factor score. First, we operationalised psychological distress as the number of symptoms present (discrete) at each time-point. This could be directly done in NCDS and BCS70 due to the use of the same instrument across cohorts and over time; and relied on three out of the seven previously harmonised symptoms that were present across all data collection points in NSHD due to the change in the version of the GHQ used in the COVID-19 Survey. Thus, the potential number of symptoms ranged from 0 to 9 in NCDS and BCS70, and from 0 to 3 in NSHD. Second, psychological distress was operationalised as 'caseness' (binary), using each of the measurement tools' recommended thresholds (**Appendix A** in this document). Finally, an additional factor approach was implemented in NSHD using the seven previously harmonised symptoms as indicators of a latent psychological distress factor.

Trajectories were modelled using different multilevel growth curve models depending on the nature of the outcome: linear models for the factor scores operationalisations (continuous), Poisson models for the number of symptoms operationalisation (discrete), and logistic models for the 'caseness' operationalisation (binary).

Analyses using NSHD data accounted for likely reporting bias during the COVID-19 survey of one of the GHQ-12 items (item 7 from GHQ-12: "Have you recently been able to enjoy your normal day to day activities"), which seemed to be more reflective of the lockdown restrictions in place than of the person's distress level (see below). This item was therefore replaced by a different item on low mood (item 9: "Have you recently been feeling unhappy or depressed?") in the approaches using harmonised symptoms.

The initial exploration of the proportion of endorsement of each of the GHQ-12 items, after recoding the responses following the GHQ-scoring system [3], showed an unusually high endorsement (51.7%) of item 7 in the COVID-19 Survey wave 1 (May 2020). This item corresponds to the question "Have you recently been able to enjoy your normal day to day activities?", which was objectively impaired considering the ongoing nationwide lockdown at the assessment time point. The difference can be clearly observed in **Figure C1**, which includes all the GHQ-12 items, with those marked with an asterisk (*) having been reversely coded to reflect psychological distress.

Since item 7 may be more reflective of the external conditions than of the psychological distress level of the individual, as most people could not legally perform their normal day to day activities back then, we were cautious when including it in the different operationalisations of psychological distress.

In the 'caseness' (binary) approach, this item may have a strong impact on the proportion of 'cases' using the recommended threshold of 2+ endorsed items, thus potentially leading to overestimation of those proportions. We compared the 'caseness' prevalence estimates resulting from three different operationalisations: a) usual approach, keeping the problematic item in and the threshold at 2+; b) dropping the item and keeping the threshold at 2+; and c) dropping the item and lowering the threshold to 1+. The weighted proportion estimates of 'caseness' at each of the three COVID-19 Survey waves is shown in **Table C1**.

Operationalisation A (usual approach) showed a very high prevalence estimate during the first survey wave, an increase that may be mostly driven by item 7 as the comparison with the other two operationalisations, which follow the inverted U-pattern, suggest. Operationalisation B showed that inverted U-pattern, consistent with what has been found in the alternative operationalisations and cohorts, and showed relatively similar results to the usual approach (operationalisation A) at survey waves 2 and 3. Operationalisation C led to the highest prevalence estimates of all operationalisations, despite showing the expected inverted U-pattern. Thus, operationalisation B was selected as the optimal approach to 'caseness' in NSHD during the COVID-19 Survey waves.

In the 'number of symptoms present' (discrete) and the factor score (continuous) approaches, which were based on the harmonisation of items mapped to specific distressful experiences, the problematic item (item 7) had been previously used as the indicator of choice for low mood [8, 9], present in the 28-item version of the GHQ used in that study. An alternative item (item 9: "Have you recently been feeling unhappy or depressed?") could be mapped to low mood in the 12-item version and was therefore used as the indicator of choice in the three COVID-19 Survey assessments (see **Appendix B** in this document).

Figure C1. Proportion of NSHD participants endorsing each GHQ-12 item in the COVID-19 Survey waves

Items with an asterisk are reversely coded

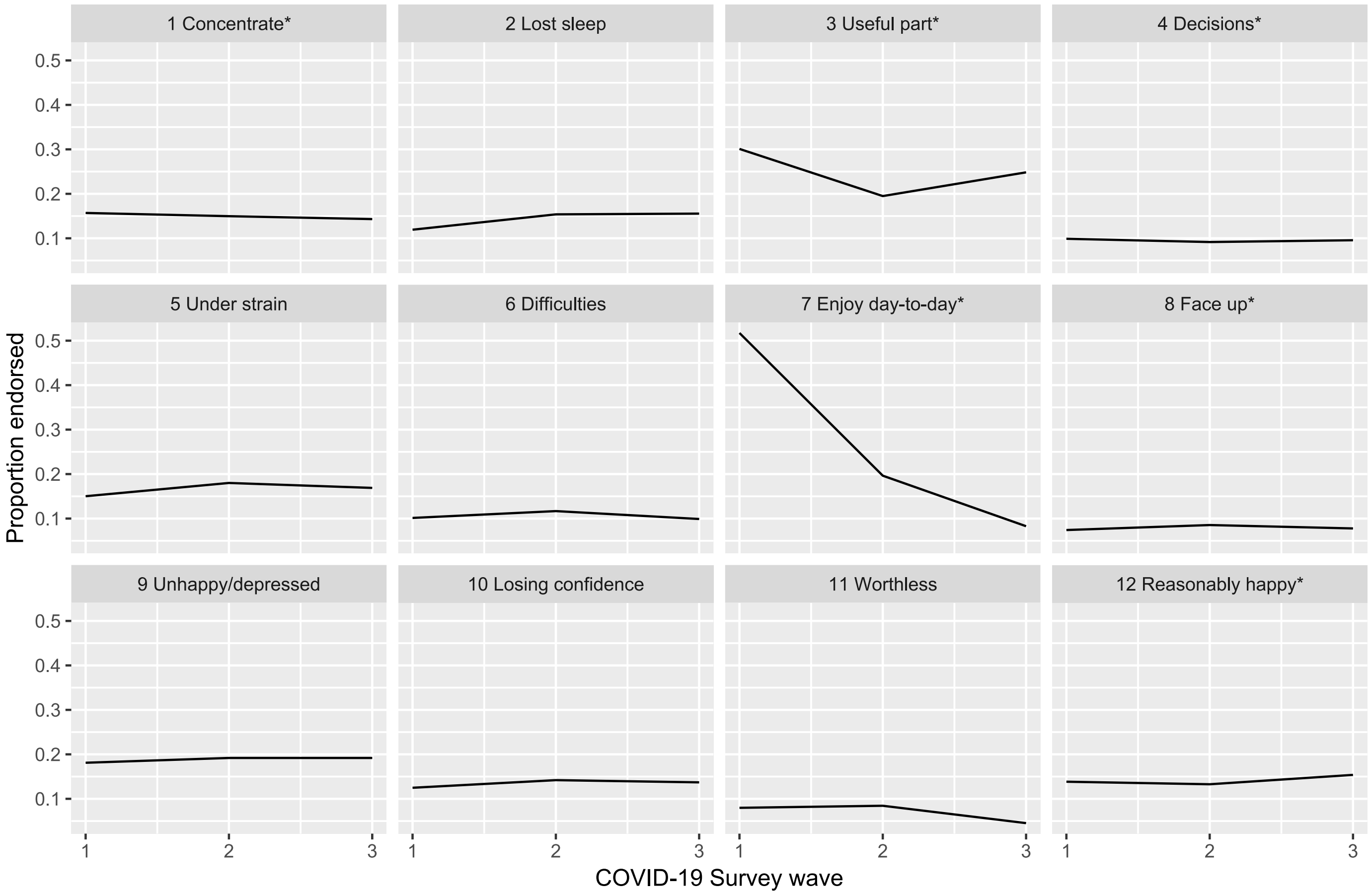

Table C1. Weighted prevalence estimates (and 95% CIs) of psychological distress 'caseness' across COVID-19 Survey waves by operationalisation

	Operationalisation A, proportion (95% CI)	Operationalisation B, proportion (95% CI)	Operationalisation C, proportion (95% CI)
Survey wave 1	0.41 [0.39, 0.44]	0.32 [0.30, 0.34]	0.49 [0.47, 0.52]
Survey wave 2	0.39 [0.37, 0.41]	0.36 [0.34, 0.38]	0.52 [0.50, 0.54]
Survey wave 3	0.29 [0.27, 0.32]	0.28 [0.26, 0.30]	0.42 [0.40, 0.44]

Note. Confidence intervals (CIs) are constructed based on the Agresti-Coull method. Operationalisation A: keeping item 7 and threshold at 2+; Operationalisation B: dropping item 7 and keeping threshold at 2+; Operationalisation C: dropping item 7 and lowering threshold to 1+.

Appendix D. Measurement invariance testing

As comparisons of the psychological distress' levels were going to be made across time points, cohorts (in the case of the models estimated using the same indicators), and sexes (in the case of the stratified models), we explored the degree of measurement invariance across these conditions where the same set of indicators were going to be used [10].

We extended the existing evidence on the invariant properties of the Malaise Inventory over time, across cohorts, and sexes [11], to include the most recent data collection points. We implemented the same procedure with the GAD-2 and PHQ-2 items to assess if they had equivalent measurement properties across the three cohorts, time-points, and sexes, as they were used as 'anchor items' in the factor score approach including the three cohorts.

More specifically, the level of invariance needed to make meaningful comparisons across conditions was different for the models using different operationalisations.

The most restrictive level of invariance was needed for the models estimated as a sensitivity check using the number of psychological distress symptoms according to the Malaise Inventory, used in the NCDS and BCS70 cohorts, as comparisons were going to be made at the observed number of symptoms (count) level, where all symptoms had the same 'weight'. Thus, not only the item loadings and thresholds had to be equivalent across conditions (scalar/strong invariance), but also the loadings should be the same across items (Rasch-type model), as each of the items were counted as a unit. Evidence of this level of invariance across time points, cohorts, and sexes had been previously obtained up to the most recent pre-pandemic assessment [9, 11], and we extended that analysis to include the three COVID-19 Survey waves.

In the factor score approach with the three cohorts, a number of indicators common across the three cohorts ('anchor' items, the GAD-2 and PHQ-2 items) were going to be used alongside the cohort- and wave-specific indicators to measure a latent psychological distress factor. Unlike the previous case, in this approach the scalar/strong invariance level would suffice, as comparisons were going to be made across factor scores, which had been obtained based on the different 'weights' (loadings) of each of the items. In this case, however, the measurement invariance testing would be limited to the 'anchor' items as a way of exploring to what extent this subset of common items was comparable across conditions (time points, cohorts, and sexes).

A structural equation modelling (SEM) framework was used, where the psychological distress latent factors were defined by their corresponding set of indicators (the nine items of the Malaise Inventory, or the GAD-2 and PHQ-2 items). Due to the nature of the indicators (categorical ordered), models were estimated using a Weighted Least Squares Mean and Variance adjusted (WLSMV) estimator and under a Delta parameterisation [12].

Models with increasing levels of constraints were estimated [10]. First, models where the same factor structure was proposed across conditions without further constraints (configural invariance model) were estimated. The fit of these configural models to the data was assessed by means of the goodness-of-fit indices, with Root Mean Square Error of Approximation (RMSEA) values close to 0.060, and Comparative Fit Index (CFI) and Tucker-Lewis Index values close to 0.950 being generally considered as indicative of good fit [13]. Then, models where both the items' loadings and thresholds were constrained to be equal across the different conditions (scalar/strong invariance model) were estimated. The goodness-of-fit indices of these models were compared with those from the configural models and deemed to be invariant (i.e., to not fit the data substantially worse) if the loss in fit was smaller than 0.010 for the CFI and 0.015 for the RMSEA [14, 15]. Finally, and only for the models with the Malaise Inventory, additional models were estimated where all items were constrained to have the same factor loading (Rasch-type model).

Based on the abovementioned criteria, scalar invariance was found to hold for the Malaise inventory in all cases tested (**Table D1**). The further constrained Rasch-type model was found to fit well the data.

In the case of the GAD-2 and PHQ-2 items, RMSEA values were found to be beyond the usual threshold for acceptable fit, whereas the CFI and TLI indices suggested good fit across the different models (**Table D2**). Due to the sensitivity of the RMSEA fit index to models with small degrees of freedom [16] (in this case, 2 degrees of freedom per group in the configural models), and considering the good performance in the alternative indices and the improvement in comparative fit with increasing constraints, we considered scalar invariance to hold.

Table D1. Malaise inventory measurement invariance testing results

	Model	Chi-square (df)	RMSEA (90% CI)	CFI	TLI	ΔRMSEA	ΔCFI
NCDS:<br>age (7: 23, 33, 42, 50, 61.7, 62, 62.5, 63) * birth sex (2)	Configural	1807 (432)	0.034 (0.032, 0.035)	0.987	0.983		
	Scalar	2837 (537)	0.039 (0.038, 0.040)	0.978	0.977	-0.005	-0.009
	**Rasch**	**3170 (545)**	**0.041 (0.040, 0.043)**	**0.975**	**0.974**	**-0.002**	**-0.003**
BCS70:<br>age (8: 26, 29, 34, 42, 46, 50, 50.5, 51) * birth sex (2)	Configural	2333 (432)	0.041 (0.039, 0.043)	0.985	0.980		
	Scalar	2962 (537)	0.041 (0.040, 0.043)	0.981	0.979	<0.001	-0.004
	**Rasch**	**3424 (545)**	**0.045 (0.043, 0.046)**	**0.977**	**0.976**	**-0.004**	**-0.004**
NCDS/BCS70:<br>age (4: 23/26, 33/34, 42, 50) * birth sex (2) * cohort (2)	Configural	2028 (432)	0.036 (0.034, 0.037)	0.985	0.980		
	Scalar	2964 (537)	0.039 (0.038, 0.041)	0.977	0.975	-0.003	-0.008
	**Rasch**	**3205 (545)**	**0.041 (0.040, 0.042)**	**0.975**	**0.973**	**-0.002**	**-0.002**

Note. BCS70: 1970 British Cohort Study; CFI: Comparative Fit Index; df: degrees of freedom; NCDS: 1958 National Child Development Study; NSHD: 1946 National Survey of Health and Development; RMSEA: Root Mean Square Error of Approximation; TLI: Tucker-Lewis Index; ΔCFI: difference in CFI; ΔRMSEA: difference in RMSEA. Selected models are highlighted in bold.

Table D2. GAD-2 and PHQ-2 measurement invariance testing results

	Model	Chi-square (df)	RMSEA (90% CI)	CFI	TLI	ΔRMSEA	ΔCFI
NSHD: CWs (3) * birth sex (2)	Configural	384 (12)	0.183 (0.167, 0.199)	0.991	0.974		
	**Scalar**	**553 (62)**	**0.092 (0.085, 0.100)**	**0.989**	**0.993**	**0.091**	**-0.002**
NCDS: CWs (3) * birth sex (2)	Configural	1402 (12)	0.200 (0.191, 0.209)	0.987	0.960		
	**Scalar**	**1881 (62)**	**0.100 (0.097, 0.104)**	**0.983**	**0.990**	**0.100**	**-0.004**
BCS70: CWs (3) * birth sex (2)	Configural	1494 (12)	0.227 (0.218, 0.237)	0.985	0.955		
	**Scalar**	**2026 (62)**	**0.115 (0.111, 0.120)**	**0.980**	**0.989**	**0.112**	**-0.005**
NSHD+NCDS+BCS70: CWs (3) * birth sex (2) * cohort (3)	Configural	3289 (36)	0.209 (0.203, 0.215)	0.987	0.961		
	**Scalar**	**4759 (206)**	**0.103 (0.101, 0.106)**	**0.982**	**0.990**	**0.106**	**-0.005**

Note. BCS70: 1970 British Cohort Study; CWs: COVID-19 Survey waves. CFI: Comparative Fit Index; df: degrees of freedom; NCDS: 1958 National Child Development Study; NSHD: 1946 National Survey of Health and Development; RMSEA: Root Mean Square Error of Approximation; TLI: Tucker-Lewis Index; ΔCFI: difference in CFI; ΔRMSEA: difference in RMSEA. Selected models are highlighted in bold.

Appendix E. STROBE checklist for cohort studies

	Item No	Recommendation	Section / paragraph
**Title and abstract**	1	(*a*) Indicate the study's design with a commonly used term in the title or the abstract	Title, Abstract
		(*b*) Provide in the abstract an informative and balanced summary of what was done and what was found	Abstract
**Introduction**			
Background/rationale	2	Explain the scientific background and rationale for the investigation being reported	Introduction / 1-2
Objectives	3	State specific objectives, including any prespecified hypotheses	Introduction / 2
**Methods**			
Study design	4	Present key elements of study design early in the paper	Methods / 1
Setting	5	Describe the setting, locations, and relevant dates, including periods of recruitment, exposure, follow-up, and data collection	Methods / 1
Participants	6	(*a*) Give the eligibility criteria, and the sources and methods of selection of participants. Describe methods of follow-up	Methods / 1
		(*b*) For matched studies, give matching criteria and number of exposed and unexposed	-
Variables	7	Clearly define all outcomes, exposures, predictors, potential confounders, and effect modifiers. Give diagnostic criteria, if applicable	Methods / 3-5<br>Appendices A-D (SM)
Data sources/ measurement	8*	For each variable of interest, give sources of data and details of methods of assessment (measurement). Describe comparability of assessment methods if there is more than one group	Methods / 3-6<br>Appendices A-D (SM)
Bias	9	Describe any efforts to address potential sources of bias	Methods / 6, 12<br>Appendices C-D (SM)
Study size	10	Explain how the study size was arrived at	Results / 1
Quantitative variables	11	Explain how quantitative variables were handled in the analyses. If applicable, describe which groupings were chosen and why	Methods / 3-4
Statistical methods	12	(*a*) Describe all statistical methods, including those used to control for confounding	Methods / 6-12<br>Appendix D (SM)
		(*b*) Describe any methods used to examine subgroups and interactions	Methods / 6, 9, 11<br>Appendix C (SM)
		(*c*) Explain how missing data were addressed	Methods / 12
		(*d*) If applicable, explain how loss to follow-up was addressed	Methods / 12
		(*e*) Describe any sensitivity analyses	Methods / 12<br>Appendix C (SM)
**Results**			
Participants	13*	(a) Report numbers of individuals at each stage of study—eg numbers potentially eligible, examined for eligibility, confirmed eligible, included in the study, completing follow-up, and analysed	Results / 1
		(b) Give reasons for non-participation at each stage	Figure 1
		(c) Consider use of a flow diagram	Figure 1
Descriptive data	14*	(a) Give characteristics of study participants (eg demographic, clinical, social) and information on exposures and potential confounders	Results / 1<br>Table 1
		(b) Indicate number of participants with missing data for each variable of interest	Appendix F (SM)
		(c) Summarise follow-up time (eg, average and total amount)	Results / 1
Outcome data	15*	Report numbers of outcome events or summary measures over time	Appendices H-K (SM)

Main results	16	(*a*) Give unadjusted estimates and, if applicable, confounder-adjusted estimates and their precision (eg, 95% confidence interval). Make clear which confounders were adjusted for and why they were included	Table 2<br><br>Appendix G (SM)
		(*b*) Report category boundaries when continuous variables were categorized	-
		(*c*) If relevant, consider translating estimates of relative risk into absolute risk for a meaningful time period	-
Other analyses	17	Report other analyses done—eg analyses of subgroups and interactions, and sensitivity analyses	Results / 5-6<br><br>Appendices H-K (SM)
**Discussion**			
Key results	18	Summarise key results with reference to study objectives	Discussion / 1
Limitations	19	Discuss limitations of the study, taking into account sources of potential bias or imprecision. Discuss both direction and magnitude of any potential bias	Discussion / 5
Interpretation	20	Give a cautious overall interpretation of results considering objectives, limitations, multiplicity of analyses, results from similar studies, and other relevant evidence	Discussion / 2-4
Generalisability	21	Discuss the generalisability (external validity) of the study results	Discussion / 5
**Other information**			
Funding	22	Give the source of funding and the role of the funders for the present study and, if applicable, for the original study on which the present article is based	Submission form

Note. SM: Supplementary Material. An Explanation and Elaboration article discusses each checklist item and gives methodological background and published examples of transparent reporting. The STROBE checklist is best used in conjunction with this article (freely available on the Web sites of PLoS Medicine at http://www.plosmedicine.org/, Annals of Internal Medicine at http://www.annals.org/, and Epidemiology at http://www.epidem.com/). Information on the STROBE Initiative is available at http://www.strobe-statement.org.

Appendix F. Number of missing observations by cohort and wave

	Age	Year	Number of missing observations
NSHD	36	1982	211
	43	1989	133
	53	1999	171
	63	2009	366
	69	2015	316
	74	May 2020	409
	74.5	Sept/Oct 2020	167
	75	Feb/Mar 2021	357
	**Age**	**Year**	**Number of missing observations**
NCDS	23	1981	1059
	33	1991	931
	42	2000	583
	50	2008	756
	61.7	Jan/Mar 2020	6196
	62	May 2020	2647
	62.5	Sept/Oct 2020	1362
	63	Feb/Mar 2021	867
	**Age**	**Year**	**Number of missing observations**
BCS70	26	1996	1678
	29	1999	867
	34	2004	1200
	42	2012	1164
	46	2016	1183
	50	May 2020	2865
	50.5	Sept/Oct 2020	1777
	51	Feb/Mar 2021	1326

Note. Number of missing observations is conditional on meeting the inclusion criteria at the time of the study: being alive and still residing in the UK and having participated in at least one COVID-19 Survey waves (May 2020, Sept/Oct 2020, or Feb/March 2021). Since each of the cohorts include data from participants born in the same week, the participants' age is homogeneous across waves/calendar years within cohorts.

Appendix G. Results from multilevel growth curve models with cross-cohort factor scores as outcome (linear models)

Table G1. Marginal mean levels predicted from the multilevel growth curve models with cross-cohort factor scores as outcome (linear models)

			All	Men	Women
	**Age**	**Year**	**Marginal mean (95% CI)**	**Marginal mean (95% CI)**	**Marginal mean (95% CI)**
**NSHD**	36	1982	-0.14 (-0.17, -0.11)	-0.21 (-0.25, -0.17)	-0.07 (-0.12, -0.03)
	43	1989	-0.03 (-0.05, 0.00)	-0.10 (-0.14, -0.07)	0.04 (0.00, 0.07)
	53	1999	0.02 (-0.01, 0.05)	-0.06 (-0.10, -0.01)	0.09 (0.05, 0.13)
	63	2009	-0.06 (-0.08, -0.03)	-0.14 (-0.17, -0.10)	0.02 (-0.02, 0.06)
	69	2015	-0.16 (-0.19, -0.13)	-0.24 (-0.29, -0.20)	-0.09 (-0.13, -0.04)
	74	May 2020	-0.08 (-0.13, -0.04)	-0.28 (-0.34, -0.22)	0.10 (0.04, 0.16)
	74.5	Sept/Oct 2020	-0.01 (-0.07, 0.05)	-0.15 (-0.23, -0.07)	0.12 (0.05, 0.20)
	75	Feb/Mar 2021	-0.04 (-0.10, 0.02)	-0.19 (-0.28, -0.10)	0.10 (0.02, 0.19)
	**Age**	**Year**	**Marginal mean (95% CI)**	**Marginal mean (95% CI)**	**Marginal mean (95% CI)**
**NCDS**	23	1981	-0.17 (-0.19, -0.16)	-0.38 (-0.40, -0.36)	0.01 (-0.01, 0.03)
	33	1991	-0.31 (-0.32, -0.29)	-0.44 (-0.46, -0.42)	-0.18 (-0.21, -0.16)
	42	2000	-0.04 (-0.06, -0.02)	-0.18 (-0.21, -0.16)	0.09 (0.07, 0.12)
	50	2008	-0.08 (-0.10, -0.06)	-0.23 (-0.26, -0.20)	0.05 (0.02, 0.08)
	61.7	Jan/Mar 2020	-0.06 (-0.09, -0.03)	-0.18 (-0.23, -0.13)	0.05 (0.00, 0.09)
	62	May 2020	-0.04 (-0.06, -0.01)	-0.23 (-0.27, -0.20)	0.14 (0.11, 0.18)
	62.5	Sept/Oct 2020	0.01 (-0.02, 0.03)	-0.20 (-0.23, -0.16)	0.20 (0.16, 0.23)
	63	Feb/Mar 2021	0.01 (-0.01, 0.04)	-0.19 (-0.22, -0.16)	0.20 (0.17, 0.23)
	**Age**	**Year**	**Marginal mean (95% CI)**	**Marginal mean (95% CI)**	**Marginal mean (95% CI)**
**BCS70**	26	1996	0.12 (0.10, 0.14)	-0.08 (-0.11, -0.05)	0.27 (0.25, 0.30)
	29	1999	0.01 (-0.01, 0.02)	-0.15 (-0.17, -0.12)	0.12 (0.10, 0.15)
	34	2004	0.01 (-0.01, 0.03)	-0.11 (-0.14, -0.08)	0.10 (0.08, 0.13)
	42	2012	0.14 (0.12, 0.16)	0.02 (-0.01, 0.05)	0.23 (0.20, 0.26)
	46	2016	0.07 (0.04, 0.09)	-0.05 (-0.09, -0.02)	0.16 (0.13, 0.19)
	50	May 2020	0.17 (0.14, 0.19)	0.00 (-0.04, 0.04)	0.30 (0.27, 0.34)
	50.5	Sept/Oct 2020	0.21 (0.19, 0.24)	0.04 (0.00, 0.08)	0.36 (0.32, 0.39)
	51	Feb/Mar 2021	0.21 (0.18, 0.23)	0.04 (-0.01, 0.08)	0.35 (0.31, 0.38)

Note. Unadjusted results. BCS70: 1970 British Cohort Study; NCDS: 1958 National Child Development Study; NSHD: 1946 National Survey of Health and Development.

Figure G1. Marginal mean cross-cohort psychological distress factor scores over time (year) by birth sex

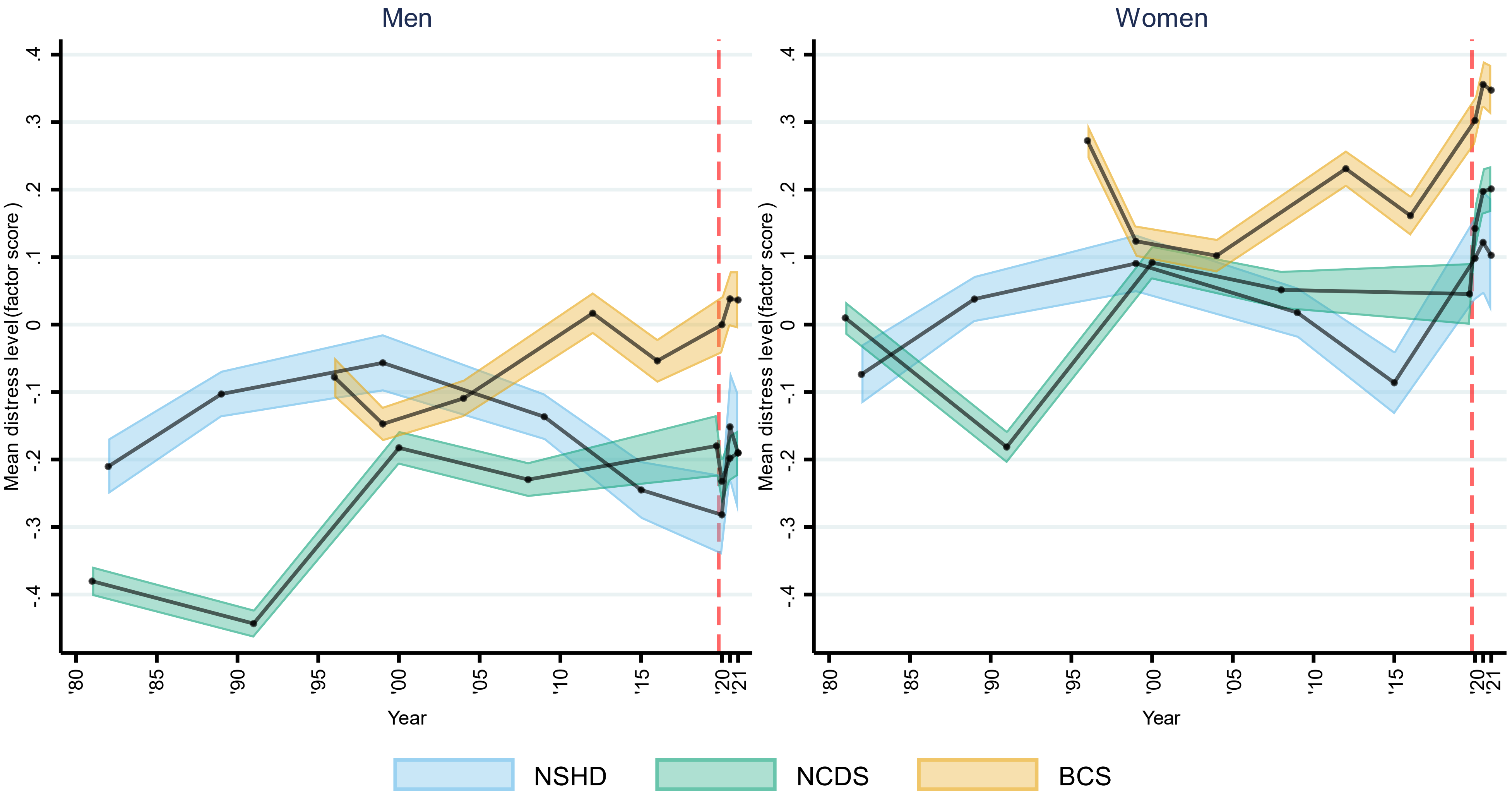

Note: Unadjusted results . 95% confidence intervals are indicated in lighter shaded areasBCS70: 1970 British Cohort Study, NCDS: 1958 National Child and Development Study; NSHD: 1946 National Survey of Health and Development .
The dashed line represents the first nationwide lockdown enforced in March 2020.

Appendix H. Results from multilevel growth curve models with cross-cohort factor scores as outcome (linear models), sensitivity checks with extended non-response weights

Table H1. Model coefficients from the multilevel growth curve models with cross-cohort factor scores as outcome (linear models), sensitivity checks with extended non-response weights

	NSHD		NCDS		BCS70	
**Models without interaction by birth sex**	**Coefficient (95% CI)**	***p***	**Coefficient (95% CI)**	***p***	**Coefficient (95% CI)**	***p***
Spline 1, linear term	0.02 (0.02, 0.02)	<0.001	-0.10 (-0.11, -0.09)	<0.001	-0.06 (-0.07, -0.05)	<0.001
Spline 1, quadratic term	-0.001 (-0.001, -0.001)	<0.001	0.013 (0.012, 0.014)	<0.001	0.007 (0.007, 0.008)	<0.001
Spline 1, cubic term			-0.0005 (-0.0006, -0.0005)	<0.001	-0.0002 (-0.0003, -0.0002)	<0.001
Spline 1, quartic term			0.00001 (0.00001, 0.00001)	<0.001		
Spline 2, linear term	-1.20 (-2.61, 0.21)	0.095	0.03 (-0.17, 0.23)	0.736	-0.42 (-0.89, 0.06)	0.085
Spline 2, quadratic term	0.44 (-0.08, 0.96)	0.095	0.14 (-0.22, 0.49)	0.448	0.20 (-0.01, 0.41)	0.068
Spline 2, cubic term	-0.04 (-0.09, 0.01)	0.101	-0.09 (-0.26, 0.07)	0.275	-0.02 (-0.04, 0.00)	0.071
Intercept	-0.14 (-0.17, -0.11)	<0.001	-0.17 (-0.19, -0.16)	<0.001	0.12 (0.10, 0.14)	<0.001
Intercept variance	0.19 (0.18, 0.21)		0.35 (0.34, 0.36)		0.40 (0.38, 0.41)	
**Models with interaction by birth sex**	**Coefficient (95% CI)**	***p***	**Coefficient (95% CI)**	***p***	**Coefficient (95% CI)**	***p***
Spline 1, linear term	0.02 (0.01, 0.03)	<0.001	-0.08 (-0.09, -0.07)	<0.001	-0.04 (-0.05, -0.03)	<0.001
Spline 1, quadratic term	-0.001 (-0.001, 0.000)	<0.001	0.012 (0.010, 0.013)	<0.001	0.006 (0.005, 0.007)	<0.001
Spline 1, cubic term			-0.0005 (-0.0005, -0.0004)	<0.001	-0.0002 (-0.0002, -0.0002)	<0.001
Spline 1, quartic term			0.00001 (0.00001, 0.00001)	<0.001		
Spline 2, linear term	-2.05 (-4.17, 0.08)	0.060	-0.30 (-0.57, -0.02)	0.035	-0.33 (-1.08, 0.42)	0.395
Spline 2, quadratic term	0.73 (-0.05, 1.52)	0.067	0.52 (0.04, 1.01)	0.035	0.15 (-0.19, 0.48)	0.383
Spline 2, cubic term	-0.06 (-0.14, 0.01)	0.076	-0.23 (-0.46, 0.00)	0.050	-0.02 (-0.05, 0.02)	0.395
Intercept * women	0.13 (0.07, 0.19)	<0.001	0.39 (0.36, 0.42)	<0.001	0.35 (0.31, 0.39)	<0.001
Spline 1, linear term * women	0.00 (-0.01, 0.01)	0.786	-0.03 (-0.05, -0.01)	<0.001	-0.03 (-0.05, -0.02)	<0.001
Spline 1, quadratic term * women	0.000 (0.000, 0.000)	0.872	0.002 (0.000, 0.004)	0.041	0.003 (0.001, 0.004)	0.007
Spline 1, cubic term * women			-0.0001 (-0.0002, 0.0000)	0.172	-0.0001 (-0.0001, 0.0000)	0.059
Spline 1, quartic term * women			0.00000 (0.00000, 0.00000)	0.333		
Spline 2, linear term * women	1.63 (-1.18, 4.43)	0.257	0.65 (0.25, 1.05)	0.001	-0.16 (-1.12, 0.80)	0.741
Spline 2, quadratic term * women	-0.56 (-1.59, 0.47)	0.288	-0.77 (-1.48, -0.06)	0.033	0.09 (-0.34, 0.51)	0.695
Spline 2, cubic term * women	0.05 (-0.05, 0.14)	0.311	0.28 (-0.06, 0.62)	0.102	-0.01 (-0.06, 0.04)	0.682
Intercept	-0.21 (-0.25, -0.17)	<0.001	-0.38 (-0.40, -0.36)	<0.001	-0.08 (-0.10, -0.05)	<0.001
Intercept variance	0.18 (0.17, 0.20)		0.33 (0.31, 0.34)		0.38 (0.36, 0.39)	

Note. Unadjusted results from sensitivity models using non-response weights at all time points. BCS70: 1970 British Cohort Study; NCDS: 1958 National Child Development Study; NSHD: 1946 National Survey of Health and Development.

Table H2. Marginal mean levels predicted from the multilevel growth curve models with cross-cohort factor scores as outcome (linear models), sensitivity checks with extended non-response weights

			All	Men	Women
	**Age**	**Year**	**Marginal mean (95% CI)**	**Marginal mean (95% CI)**	**Marginal mean (95% CI)**
**NSHD**	36	1982	-0.14 (-0.17, -0.11)	-0.21 (-0.25, -0.17)	-0.07 (-0.12, -0.03)
	43	1989	-0.03 (-0.05, 0.00)	-0.10 (-0.14, -0.07)	0.04 (0.01, 0.07)
	53	1999	0.02 (-0.01, 0.05)	-0.06 (-0.10, -0.01)	0.09 (0.05, 0.14)
	63	2009	-0.06 (-0.08, -0.03)	-0.14 (-0.17, -0.10)	0.02 (-0.02, 0.05)
	69	2015	-0.16 (-0.20, -0.13)	-0.24 (-0.29, -0.20)	-0.09 (-0.14, -0.04)
	74	May 2020	-0.09 (-0.13, -0.04)	-0.29 (-0.35, -0.23)	0.10 (0.03, 0.16)
	74.5	Sept/Oct 2020	-0.01 (-0.07, 0.05)	-0.15 (-0.24, -0.07)	0.12 (0.05, 0.20)
	75	Feb/Mar 2021	-0.04 (-0.10, 0.02)	-0.19 (-0.28, -0.10)	0.10 (0.02, 0.19)
	**Age**	**Year**	**Marginal mean (95% CI)**	**Marginal mean (95% CI)**	**Marginal mean (95% CI)**
**NCDS**	23	1981	-0.17 (-0.19, -0.16)	-0.38 (-0.40, -0.36)	0.01 (-0.01, 0.04)
	33	1991	-0.31 (-0.32, -0.29)	-0.44 (-0.46, -0.42)	-0.18 (-0.20, -0.16)
	42	2000	-0.04 (-0.06, -0.02)	-0.18 (-0.21, -0.16)	0.09 (0.07, 0.12)
	50	2008	-0.08 (-0.10, -0.06)	-0.23 (-0.25, -0.20)	0.05 (0.03, 0.08)
	61.7	Jan/Mar 2020	-0.06 (-0.10, -0.03)	-0.18 (-0.23, -0.14)	0.05 (0.00, 0.09)
	62	May 2020	-0.04 (-0.06, -0.01)	-0.23 (-0.27, -0.20)	0.14 (0.10, 0.18)
	62.5	Sept/Oct 2020	0.01 (-0.02, 0.03)	-0.20 (-0.23, -0.16)	0.20 (0.17, 0.23)
	63	Feb/Mar 2021	0.01 (-0.02, 0.03)	-0.20 (-0.23, -0.16)	0.20 (0.16, 0.23)
	**Age**	**Year**	**Marginal mean (95% CI)**	**Marginal mean (95% CI)**	**Marginal mean (95% CI)**
**BCS70**	26	1996	0.12 (0.10, 0.14)	-0.08 (-0.10, -0.05)	0.28 (0.25, 0.30)
	29	1999	0.01 (-0.01, 0.02)	-0.14 (-0.17, -0.12)	0.13 (0.10, 0.15)
	34	2004	0.01 (-0.01, 0.03)	-0.11 (-0.14, -0.08)	0.10 (0.08, 0.13)
	42	2012	0.14 (0.12, 0.16)	0.02 (-0.01, 0.05)	0.23 (0.20, 0.26)
	46	2016	0.07 (0.05, 0.09)	-0.05 (-0.09, -0.02)	0.16 (0.13, 0.19)
	50	May 2020	0.17 (0.14, 0.19)	0.00 (-0.04, 0.04)	0.30 (0.27, 0.34)
	50.5	Sept/Oct 2020	0.21 (0.18, 0.24)	0.03 (-0.01, 0.08)	0.35 (0.32, 0.39)
	51	Feb/Mar 2021	0.20 (0.18, 0.23)	0.04 (-0.01, 0.08)	0.34 (0.31, 0.38)

Note. Unadjusted results from sensitivity models using non-response weights at all time points. BCS70: 1970 British Cohort Study; NCDS: 1958 National Child Development Study; NSHD: 1946 National Survey of Health and Development.

Appendix I. Results from multilevel growth curve models with number of symptoms as outcome (Poisson models)

Table I1. Model coefficients from the multilevel growth curve models with number of symptoms as outcome (Poisson models)

	NSHD			NCDS			BCS70	
**Models without interaction by birth sex**	**Coefficient (95% CI)**	***p***		**Coefficient (95% CI)**	***p***		**Coefficient (95% CI)**	***p***
Spline 1, linear term	0.05 (0.03, 0.06)	<0.001		-0.17 (-0.18, -0.15)	<0.001		-0.06 (-0.07, -0.05)	<0.001
Spline 1, quadratic term	-0.002 (-0.002, -0.001)	<0.001		0.022 (0.020, 0.024)	<0.001		0.008 (0.007, 0.009)	<0.001
Spline 1, cubic term				-0.0008 (-0.0009, -0.0008)	<0.001		-0.0003 (-0.0003, -0.0002)	<0.001
Spline 1, quartic term				0.00001 (0.00001, 0.00001)	<0.001			
Spline 2, linear term	-1.31 (-7.14, 4.52)	0.659		-0.35 (-0.65, -0.04)	0.025		-1.86 (-2.38, -1.34)	<0.001
Spline 2, quadratic term	0.52 (-1.64, 2.68)	0.635		1.08 (0.56, 1.59)	<0.001		0.83 (0.60, 1.06)	<0.001
Spline 2, cubic term	-0.05 (-0.25, 0.15)	0.639		-0.59 (-0.82, -0.35)	<0.001		-0.09 (-0.12, -0.07)	<0.001
Intercept	-1.70 (-1.82, -1.59)	<0.001		-0.44 (-0.48, -0.40)	<0.001		0.13 (0.10, 0.16)	<0.001
Intercept variance	1.60 (1.44, 1.77)			1.24 (1.18, 1.30)			0.96 (0.91, 1.00)	
**Models with interaction by birth sex**	**Coefficient (95% CI)**	***p***		**Coefficient (95% CI)**	***p***		**Coefficient (95% CI)**	***p***
Spline 1, linear term	0.06 (0.04, 0.08)	<0.001		-0.19 (-0.22, -0.16)	<0.001		-0.05 (-0.07, -0.03)	<0.001
Spline 1, quadratic term	-0.002 (-0.003, -0.001)	<0.001		0.027 (0.023, 0.030)	<0.001		0.008 (0.005, 0.010)	<0.001
Spline 1, cubic term				-0.0011 (-0.0012, -0.0009)	<0.001		-0.0002 (-0.0003, -0.0002)	<0.001
Spline 1, quartic term				0.00001 (0.00001, 0.00002)	<0.001			<0.001
Spline 2, linear term	-8.51 (-14.66, -2.36)	0.007		-0.56 (-1.07, -0.06)	0.029		-1.67 (-2.64, -0.69)	0.001
Spline 2, quadratic term	3.16 (0.89, 5.42)	0.006		1.27 (0.39, 2.15)	0.005		0.73 (0.30, 1.17)	0.001
Spline 2, cubic term	-0.29 (-0.50, -0.08)	0.007		-0.64 (-1.05, -0.23)	0.002		-0.08 (-0.13, -0.03)	0.001
Intercept * women	0.53 (0.32, 0.74)	<0.001		0.90 (0.82, 0.97)	<0.001		0.57 (0.51, 0.64)	<0.001
Spline 1, linear term * women	-0.02 (-0.05, 0.01)	0.176		0.04 (0.00, 0.07)	0.036		-0.02 (-0.05, 0.00)	0.036
Spline 1, quadratic term * women	0.001 (0.000, 0.002)	0.077		-0.008 (-0.012, -0.004)	<0.001		0.001 (-0.002, 0.003)	0.557
Spline 1, cubic term * women				0.0004 (0.0002, 0.0005)	<0.001		0.0000 (-0.0001, 0.0001)	0.982
Spline 1, quartic term * women				0.00000 (-0.00001, 0.00000)	<0.001			<0.001
Spline 2, linear term * women	11.06 (1.12, 21.00)	0.029		0.37 (-0.26, 1.00)	0.253		-0.26 (-1.40, 0.87)	0.651
Spline 2, quadratic term * women	-4.05 (-7.72, -0.37)	0.031		-0.34 (-1.42, 0.75)	0.546		0.13 (-0.37, 0.64)	0.601
Spline 2, cubic term * women	0.37 (0.03, 0.71)	0.032		0.10 (-0.41, 0.61)	0.700		-0.02 (-0.07, 0.04)	0.583
Intercept	-1.98 (-2.16, -1.80)	<0.001		-0.95 (-1.02, -0.88)	<0.001		-0.20 (-0.26, -0.15)	<0.001
Intercept variance	1.51 (1.36, 1.67)			1.13 (1.08, 1.18)			0.90 (0.86, 0.95)	

Note. Unadjusted results. BCS70: 1970 British Cohort Study; NCDS: 1958 National Child Development Study; NSHD: 1946 National Survey of Health and Development.

Table I2. Marginal mean levels predicted from the multilevel growth curve models with number of symptoms as outcome (Poisson models)

	Age	Year	Marginal mean (95% CI)
**NSHD**	36	1982	0.40 (0.37, 0.44)
	43	1989	0.52 (0.48, 0.55)
	53	1999	0.55 (0.51, 0.60)
	63	2009	0.42 (0.39, 0.46)
	69	2015	0.31 (0.27, 0.34)
	74	May 2020	0.54 (0.48, 0.60)
	74.5	Sept/Oct 2020	0.60 (0.49, 0.72)
	75	Feb/Mar 2021	0.60 (0.47, 0.73)
	**Age**	**Year**	**Marginal mean (95% CI)**
**NCDS**	23	1981	1.19 (1.15, 1.23)
	33	1991	0.96 (0.92, 1.00)
	42	2000	1.55 (1.51, 1.60)
	50	2008	1.51 (1.46, 1.56)
	61.7	Jan/Mar 2020	1.51 (1.42, 1.59)
	62	May 2020	1.48 (1.42, 1.54)
	62.5	Sept/Oct 2020	1.70 (1.64, 1.76)
	63	Feb/Mar 2021	1.61 (1.55, 1.67)
	**Age**	**Year**	**Marginal mean (95% CI)**
**BCS70**	26	1996	1.84 (1.79, 1.89)
	29	1999	1.63 (1.58, 1.67)
	34	2004	1.65 (1.60, 1.69)
	42	2012	1.95 (1.89, 2.00)
	46	2016	1.85 (1.79, 1.90)
	50	May 2020	1.88 (1.81, 1.94)
	50.5	Sept/Oct 2020	2.14 (2.08, 2.21)
	51	Feb/Mar 2021	2.00 (1.94, 2.07)

Note. Unadjusted results. BCS70: 1970 British Cohort Study; NCDS: 1958 National Child Development Study; NSHD: 1946 National Survey of Health and Development.

Figure I1. Marginal mean number of psychological distress symptoms over time (year and age)

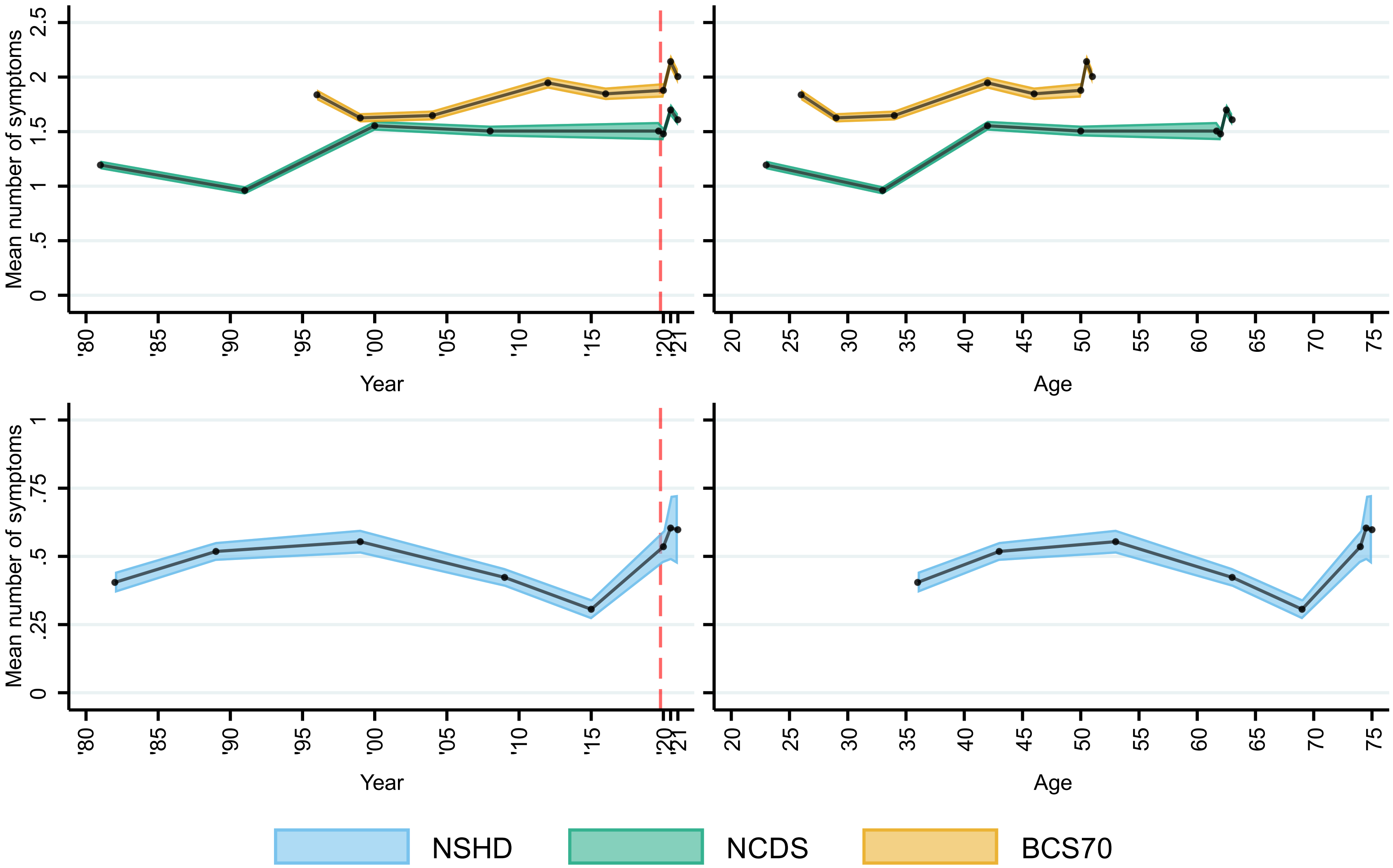

Note: Unadjusted results. 95% confidence intervals are indicated in lighter shaded areas . BCS70: 1970 British Cohort Study; NCDS: 1958 National Child and Development Study; NSHD: 1946 National Survey of Health and Development . Y axes are different for NCDS/BCS70 and NSHD as they reflect different total possible numbers of symptoms .
The dashed line represents the first nationwide lockdown enforced in March 2020.

Appendix J. Results from multilevel growth curve models with caseness as outcome (logistic models)

Table J1. Model coefficients from the multilevel growth curve models with caseness as outcome (logistic models)

	NSHD			NCDS			BCS70	
**Models without interaction by birth sex**	**Coefficient (95% CI)**	***p***		**Coefficient (95% CI)**	***p***		**Coefficient (95% CI)**	***p***
Spline 1, linear term	0.18 (0.14, 0.21)	<0.001		-0.30 (-0.38, -0.23)	<0.001		-0.14 (-0.19, -0.08)	<0.001
Spline 1, quadratic term	-0.004 (-0.005, -0.004)	<0.001		0.039 (0.029, 0.049)	<0.001		0.017 (0.011, 0.024)	<0.001
Spline 1, cubic term	0.00 (0.00, 0.00)	<0.001		-0.0014 (-0.0018, -0.0010)	<0.001		-0.0005 (-0.0007, -0.0003)	<0.001
Spline 1, quartic term	0.00 (0.00, 0.00)	<0.001		0.00002 (0.00001, 0.00002)	<0.001		0.00 (0.00, 0.00)	<0.001
Spline 2, linear term	-2.60 (-13.45, 8.24)	0.638		-1.62 (-3.06, -0.18)	0.028		-5.68 (-8.30, -3.07)	<0.001
Spline 2, quadratic term	1.21 (-2.80, 5.22)	0.555		4.08 (1.61, 6.54)	0.001		2.50 (1.34, 3.66)	<0.001
Spline 2, cubic term	-0.12 (-0.49, 0.25)	0.521		-2.06 (-3.20, -0.92)	<0.001		-0.27 (-0.40, -0.14)	<0.001
Intercept	-4.52 (-4.86, -4.19)	<0.001		-4.69 (-4.89, -4.49)	<0.001		-3.27 (-3.41, -3.12)	<0.001
Intercept variance	5.05 (4.44, 5.75)			7.66 (6.94, 8.45)			6.87 (6.32, 7.47)	
**Models with interaction by birth sex**	**Coefficient (95% CI)**	***p***		**Coefficient (95% CI)**	***p***		**Coefficient (95% CI)**	***p***
Spline 1, linear term	0.20 (0.15, 0.26)	<0.001		-0.32 (-0.47, -0.18)	<0.001		-0.11 (-0.21, -0.02)	0.017
Spline 1, quadratic term	-0.005 (-0.007, -0.004)	<0.001		0.049 (0.031, 0.067)	<0.001		0.018 (0.008, 0.029)	0.001
Spline 1, cubic term	0.00 (0.00, 0.00)	<0.001		-0.0019 (-0.0026, -0.0012)	<0.001		-0.0005 (-0.0009, -0.0002)	0.002
Spline 1, quartic term	0.00 (0.00, 0.00)	<0.001		0.00002 (0.00001, 0.00003)	<0.001		0.00 (0.00, 0.00)	<0.001
Spline 2, linear term	-17.57 (-30.64, -4.51)	0.008		-2.91 (-5.26, -0.56)	0.015		-5.26 (-9.99, -0.54)	0.029
Spline 2, quadratic term	6.70 (1.88, 11.53)	0.006		5.22 (1.15, 9.30)	0.012		2.27 (0.18, 4.36)	0.034
Spline 2, cubic term	-0.62 (-1.07, -0.18)	0.006		-2.37 (-4.27, -0.46)	0.015		-0.24 (-0.47, -0.01)	0.038
Intercept * women	1.29 (0.68, 1.91)	<0.001		2.02 (1.68, 2.36)	<0.001		1.23 (0.97, 1.50)	<0.001
Spline 1, linear term * women	-0.04 (-0.12, 0.03)	0.237		0.04 (-0.13, 0.21)	0.621		-0.03 (-0.14, 0.08)	0.599
Spline 1, quadratic term * women	0.001 (-0.001, 0.003)	0.162		-0.016 (-0.037, 0.005)	0.141		-0.002 (-0.015, 0.011)	0.769
Spline 1, cubic term * women	0.00 (0.00, 0.00)	<0.001		0.0008 (-0.0001, 0.0017)	0.071		0.0001 (-0.0003, 0.0005)	0.657
Spline 1, quartic term * women	0.00 (0.00, 0.00)	<0.001		-0.00001 (-0.00002, 0.00000)	0.047		0.00 (0.00, 0.00)	<0.001
Spline 2, linear term * women	26.80 (7.61, 45.98)	0.006		2.15 (-0.82, 5.12)	0.156		-0.65 (-6.27, 4.98)	0.822
Spline 2, quadratic term * women	-9.83 (-16.92, -2.74)	0.007		-1.98 (-7.09, 3.14)	0.448		0.36 (-2.13, 2.86)	0.774
Spline 2, cubic term * women	0.90 (0.24, 1.55)	0.007		0.55 (-1.83, 2.93)	0.650		-0.04 (-0.32, 0.23)	0.758
Intercept	-5.25 (-5.77, -4.72)	<0.001		-5.87 (-6.20, -5.54)	<0.001		-4.00 (-4.24, -3.76)	<0.001
Intercept variance	4.79 (4.20, 5.47)			7.06 (6.39, 7.80)			6.67 (6.13, 7.26)	

Note. Unadjusted results. BCS70: 1970 British Cohort Study; NCDS: 1958 National Child Development Study; NSHD: 1946 National Survey of Health and Development.

Table J2. Marginal mean levels predicted from the multilevel growth curve models with caseness as outcome (logistic models)

	Age	Year	Marginal mean (95% CI)
NSHD	36	1982	0.06 (0.05, 0.07)
	43	1989	0.11 (0.10, 0.12)
	53	1999	0.16 (0.15, 0.18)
	63	2009	0.14 (0.13, 0.16)
	69	2015	0.10 (0.09, 0.12)
	74	May 2020	0.30 (0.27, 0.32)
	74.5	Sept/Oct 2020	0.30 (0.26, 0.35)
	75	Feb/Mar 2021	0.27 (0.22, 0.31)
	**Age**	**Year**	**Marginal mean (95% CI)**
NCDS	23	1981	0.08 (0.07, 0.08)
	33	1991	0.06 (0.06, 0.07)
	42	2000	0.12 (0.11, 0.12)
	50	2008	0.14 (0.13, 0.15)
	61.7	Jan/Mar 2020	0.13 (0.12, 0.15)
	62	May 2020	0.12 (0.11, 0.13)
	62.5	Sept/Oct 2020	0.16 (0.15, 0.17)
	63	Feb/Mar 2021	0.15 (0.14, 0.16)
	**Age**	**Year**	**Marginal mean (95% CI)**
BCS70	26	1996	0.15 (0.14, 0.16)
	29	1999	0.13 (0.13, 0.14)
	34	2004	0.14 (0.13, 0.14)
	42	2012	0.18 (0.17, 0.19)
	46	2016	0.19 (0.18, 0.20)
	50	May 2020	0.18 (0.17, 0.19)
	50.5	Sept/Oct 2020	0.22 (0.21, 0.23)
	51	Feb/Mar 2021	0.20 (0.19, 0.22)

Note. Unadjusted results. BCS70: 1970 British Cohort Study; NCDS: 1958 National Child Development Study; NSHD: 1946 National Survey of Health and Development.

Figure J1. Marginal predicted mean probability of psychological distress over time (year and age)

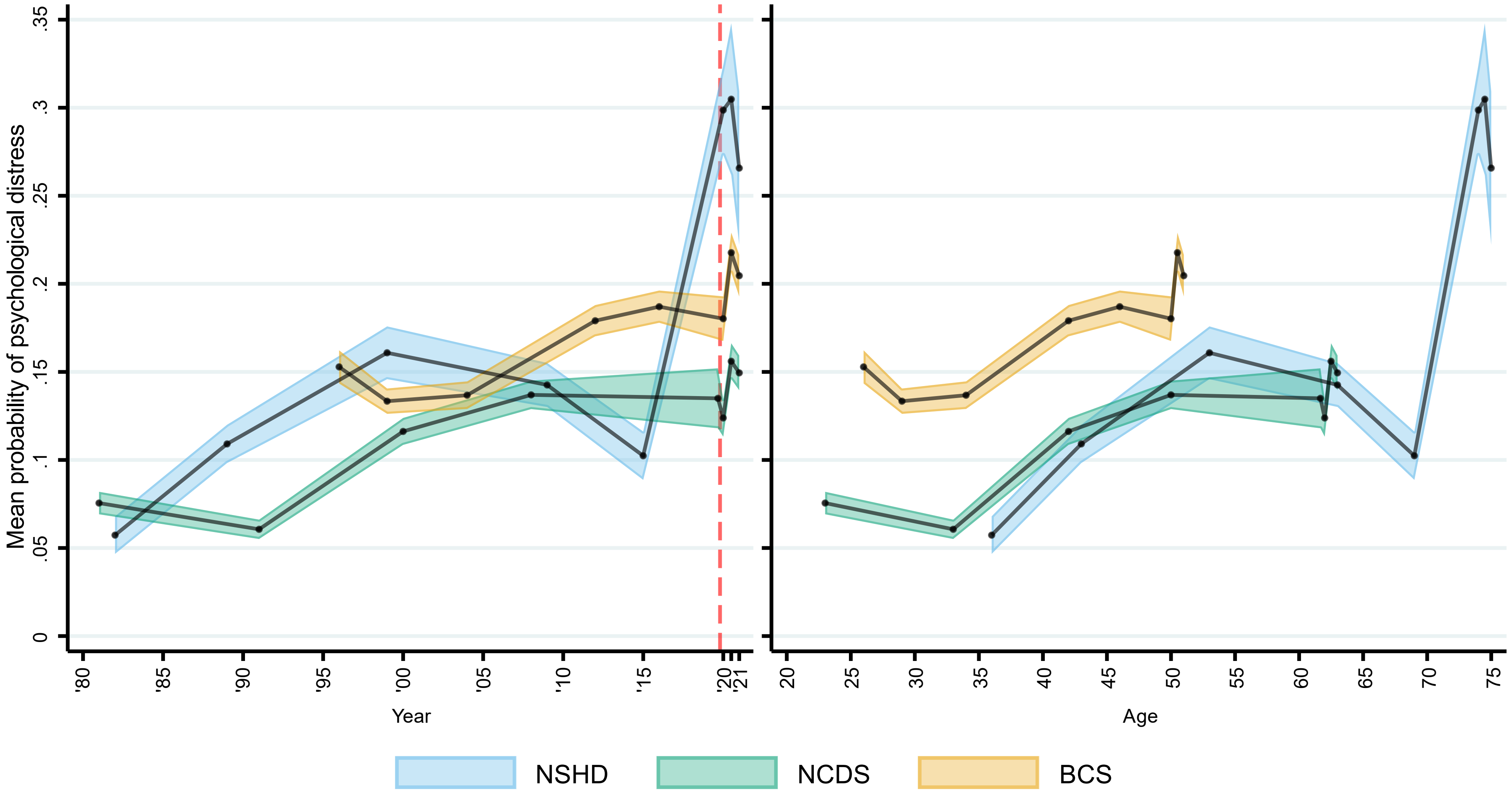

Note: Unadjusted results . 95% confidence intervals are indicated in lighter shaded areasBCS70: 1970 British Cohort Study, NCDS: 1958 National Child and Development Study; NSHD: 1946 National Survey of Health and Development .
The dashed line represents the first nationwide lockdown enforced in March 2020.

Appendix K. Results from multilevel growth curve models with factor scores as outcome (linear models), 7 harmonised psychological distress indicators in NSHD

Table K1. Model coefficients from the multilevel growth curve models with factor scores as outcome (linear models), 7 harmonised indicators in NSHD

Models without interaction by birth sex	Coefficient (95% CI)	*p*
Spline 1, linear term	0.05 (0.04, 0.07)	<0.001
Spline 1, quadratic term	-0.002 (-0.002, -0.001)	<0.001
Spline 2, linear term	-2.17 (-9.18, 4.83)	0.543
Spline 2, quadratic term	0.84 (-1.76, 3.43)	0.527
Spline 2, cubic term	-0.08 (-0.31, 0.16)	0.530
Intercept	-0.25 (-0.34, -0.16)	<0.001
Intercept variance	1.23 (1.12, 1.35)	
**Models with interaction by birth sex**	**Coefficient (95% CI)**	***p***
Spline 1, linear term	0.05 (0.04, 0.07)	<0.001
Spline 1, quadratic term	-0.002 (-0.002, -0.001)	<0.001
Spline 2, linear term	-8.22 (-14.46, -1.97)	0.010
Spline 2, quadratic term	3.04 (0.74, 5.35)	0.010
Spline 2, cubic term	-0.28 (-0.49, -0.07)	0.010
Intercept * women	0.40 (0.22, 0.57)	<0.001
Spline 1, linear term * women	0.00 (-0.03, 0.03)	0.943
Spline 1, quadratic term * women	0.000 (-0.001, 0.001)	0.860
Spline 2, linear term * women	12.06 (-1.41, 25.53)	0.079
Spline 2, quadratic term * women	-4.41 (-9.39, 0.58)	0.083
Spline 2, cubic term * women	0.40 (-0.06, 0.86)	0.086
Intercept	-0.46 (-0.58, -0.34)	<0.001
Intercept variance	1.17 (1.06, 1.29)	

Note. Unadjusted results.

Table K2. Marginal mean levels predicted from the multilevel growth curve models with factor scores as outcome (linear models), 7 harmonised indicators in NSHD

Age	Year	Marginal mean (95% CI)
36	1982	-0.25 (-0.34, -0.16)
43	1989	0.05 (-0.02, 0.12)
53	1999	0.17 (0.08, 0.25)
63	2009	-0.07 (-0.14, 0.01)
69	2015	-0.38 (-0.47, -0.29)
74	May 2020	0.09 (-0.03, 0.22)
74.5	Sept/Oct 2020	0.23 (-0.01, 0.47)
75	Feb/Mar 2021	0.16 (-0.09, 0.40)

Note. Unadjusted results.

Figure K1. Marginal mean psychological distress factor scores over time (year), 7 harmonised psychological distress indicators in NSHD

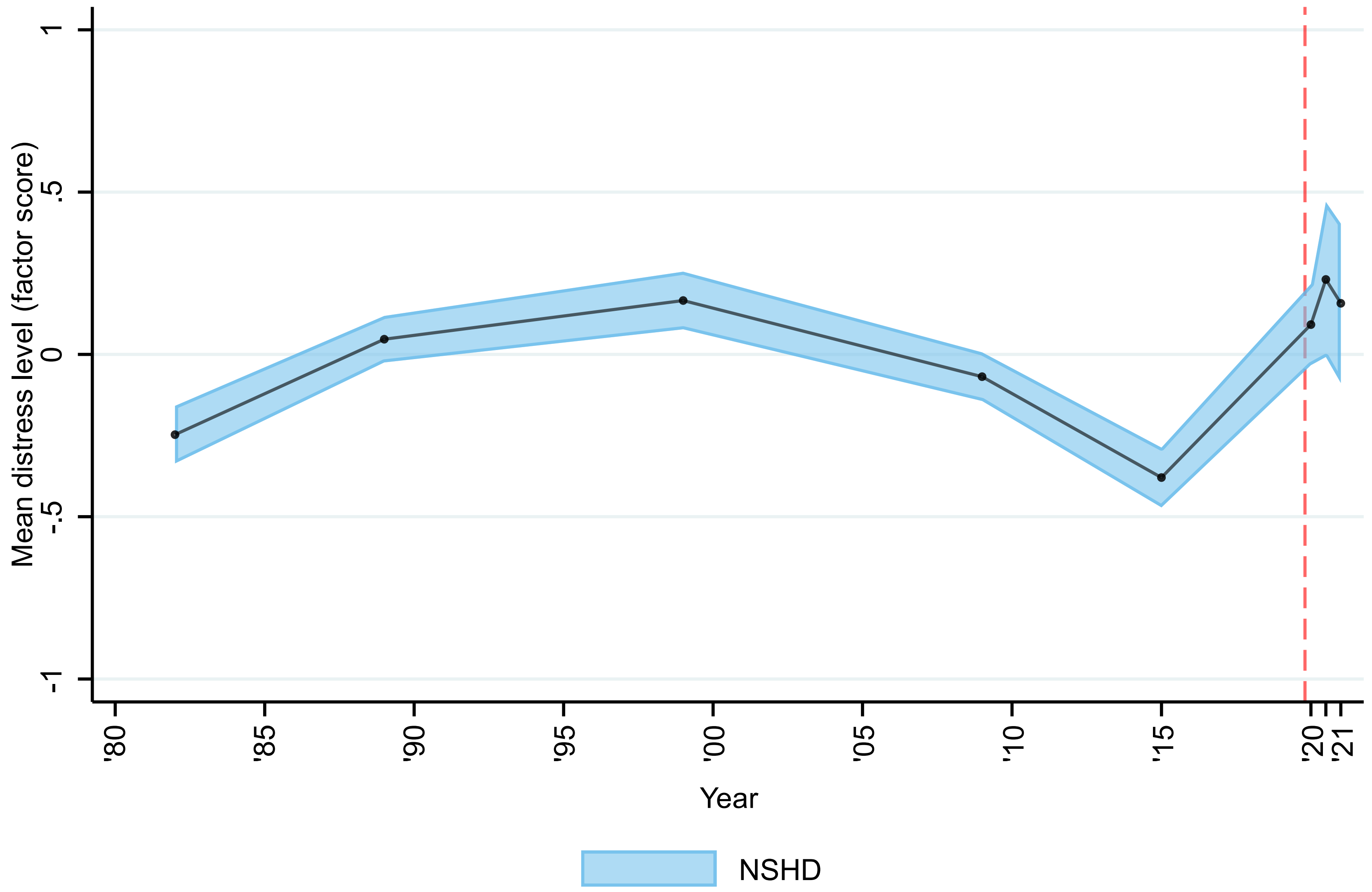

Note: Unadjusted results. 95% confidence intervals are indicated in lighter shaded areas. NSHD: 1946 National Survey of Health and Development. The dashed line represents the first nationwide lockdown enforced in March 2020.

Additional references

1. Wing JK, Cooper JE, Sartorius N. Measurement and classification of psychiatric symptoms: An instruction manual for the PSE and CATEGO program: Cambridge University Press; 1974.

2. Lindelow M, Hardy R, Rodgers B. Development of a scale to measure symptoms of anxiety and depression in the general UK population: the psychiatric symptom frequency scale. J Epidemiol Community Health. 1997;51(5):549-57. doi: 10.1136/jech.51.5.549.

3. Goldberg DP, Gater R, Sartorius N, Ustun TB, Piccinelli M, Gureje O, et al. The validity of two versions of the GHQ in the WHO study of mental illness in general health care. Psychol Med. 1997;27(1):191-7. doi: 10.1017/s0033291796004242.

4. Rodgers B, Pickles A, Power C, Collishaw S, Maughan B. Validity of the Malaise Inventory in general population samples. Soc Psychiatry Psychiatr Epidemiol. 1999;34(6):333-41. doi: 10.1007/s001270050153.

5. Ploubidis GB, Sullivan A, Brown M, Goodman A. Psychological distress in mid-life: evidence from the 1958 and 1970 British birth cohorts. Psychol Med. 2017;47(2):291-303. doi: 10.1017/S0033291716002464.

6. Kroenke K, Spitzer RL, Williams JB. The Patient Health Questionnaire-2: validity of a two-item depression screener. Med Care. 2003;41(11):1284-92. doi: 10.1097/01.MLR.0000093487.78664.3C.

7. Kroenke K, Spitzer RL, Williams JB, Monahan PO, Lowe B. Anxiety disorders in primary care: prevalence, impairment, comorbidity, and detection. Ann Intern Med. 2007;146(5):317-25. doi: 10.7326/0003-4819-146-5-200703060-00004.

8. Gondek D, Bann D, Patalay P, Goodman A, McElroy E, Richards M, et al. Psychological distress from early adulthood to early old age: evidence from the 1946, 1958 and 1970 British birth cohorts. Psychol Med. 2021:1-10. doi: 10.1017/S003329172000327X.

9. McElroy E, Villadsen A, Patalay P, Goodman A, Richards M, Northstone K, et al. Harmonisation and Measurement Properties of Mental Health Measures in Six British Cohorts. London: CLOSER; 2020.

10. Meredith W. Measurement invariance, factor analysis and factorial invariance. Psychometrika. 1993;58(4):525-43. doi: 10.1007/BF02294825.

11. Ploubidis GB, McElroy E, Moreira HC. A longitudinal examination of the measurement equivalence of mental health assessments in two British birth cohorts. Longit Life Course Stud. 2019;10(4):471-89. doi: 10.1332/175795919X15683588979486.

12. Muthen B, Asparouhov T. Latent variable analysis with categorical outcomes: multiple-group and growth modeling in Mplus. Mplus Web Notes. 2002;4(5):1-22.

13. Hu Lt, Bentler PM. Cutoff criteria for fit indexes in covariance structure analysis: Conventional criteria versus new alternatives. Struct Equ Modeling. 1999;6(1):1-55. doi: 10.1080/10705519909540118.

14. Chen FF. Sensitivity of Goodness of Fit Indexes to Lack of Measurement Invariance. Struct Equ Modeling. 2007;14(3):464-504. doi: 10.1080/10705510701301834.

15. Cheung GW, Rensvold RB. Evaluating goodness-of-fit indexes for testing measurement invariance. Struct Equ Modeling. 2002;9(2):233-55. doi: 10.1207/s15328007sem0902_5.

16. Kenny DA, McCoach DB. Effect of the Number of Variables on Measures of Fit in Structural Equation Modeling. Struct Equ Modeling. 2003;10(3):333-51. doi: 10.1207/S15328007SEM1003_1.
