# Supplementary material for: Long-term psychological distress trajectories and the COVID-19 pandemic in three British birth cohorts: A multi-cohort study
Source: PLoS Med. 2023 Apr 4;20(4):e1004145. doi: 10.1371/journal.pmed.1004145 (PMC10072377; doi:10.1371/journal.pmed.1004145)
Supplement: Attachment — Submitted filename: Response to reviewers.pdf [file pmed.1004145.s002.pdf]

September 21, 2022

Prof Vikram Patel, Prof Toshi A Furukawa,
Prof Lola Kola, and Prof Daisy Fancourt

Guest Editors, *PLOS Medicine*

Dear Professors Patel, Furukawa, Kola, and Fancourt,

We really appreciate the comments made by the editors and reviewers on the manuscript "Disruption of long-term psychological distress trajectories during the COVID-19 pandemic: evidence from three British birth cohorts" (reference: PMEDICINE-D-22-01955R1), submitted to the *PLOS Medicine* call for papers on *COVID-19 Pandemic and Global Mental Health*.

We were pleased that the reviewers considered our article interesting and relevant. We have revised the manuscript to address the suggestions that the editors and reviewers provided, and we believe that the manuscript is stronger as a result. Please find our responses to the specific points raised by the editors and reviewers below:

Editors.

Comment 1.

Please revise your title according to PLOS Medicine's style. Your title must be nondeclarative and should include the study design ("A cohort study") in the subtitle. I would suggest "Associations between long-term psychological distress trajectories and the COVID-19 pandemic in the UK: a cohort study", or similar.

Response 1.

We have changed the title to "Long-term psychological distress trajectories and the COVID-19 pandemic in three British birth cohorts: a multi-cohort study". We have also included a short title as per the submission guidelines.

Comment 2.

Please structure your abstract using the PLOS Medicine headings (Background, Methods and Findings, Conclusions).

Response 2.

We have changed the abstract structure accordingly.

Comment 3.

Abstract Methods and Findings:

* Please include the years during which the study took place, length of follow up, and main outcome measures.

* Please quantify the main results (with 95% CIs and p values).

* Please include the important dependent variables that are adjusted for in the analyses.

* In the last sentence of the Abstract Methods and Findings section, please describe the main limitation(s) of the study's methodology.

Response 3.

Thank you for this suggestion. We have updated the Abstract to include all the suggested information:

> ***In text (lines 34-68):***
>
> **Background.** Growing evidence suggests that population mental health outcomes have worsened since the pandemic started. The extent that these changes have altered common age-related trends in psychological distress, where distress typically rises until midlife and then falls after midlife in both sexes, is unknown. We aimed to analyse whether long-term pre-pandemic psychological distress trajectories were disrupted during the pandemic, and whether these changes have been different across cohorts and by sex.
>
> **Methods and Findings.** We used data from three nationally representative birth cohorts comprising all people born in Great Britain in a single week of 1946 (National Survey of Health and Development, NSHD), 1958 (National Child Development Study, NCDS), or 1970 (British Cohort Study, BCS70). The follow-up data used spanned 39 years in NSHD (1982-2021), 40 years in NCDS (1981-2001), and 25 years in BCS70 (1996-2021). We used psychological distress factor scores, as measured by validated self-reported questionnaires (NSHD:

Present State Examination, Psychiatric Symptoms Frequency, and 28- and 12-item versions of General Health Questionnaire; NCDS and BCS70: Malaise Inventory; all: 2-item versions of Generalized Anxiety Disorder scale and Patient Health Questionnaire). We used a multilevel growth curve modelling approach to model the trajectories of distress across cohorts and sexes and obtained estimates of the differences between the distress levels observed during the pandemic and those observed at the most recent pre-pandemic assessment and at the peak in the cohort-specific pre-pandemic distress trajectory, located at midlife. We further analysed whether pre-existing cohort and sex inequalities had changed with the pandemic onset using a Difference-in-Differences approach. The analytic sample included 16,389 participants. By September/October 2020, distress levels had reached or exceeded the levels of the peak in the pre-pandemic life-course trajectories, with larger increases in younger cohorts (Standardised Mean Differences and 95% confidence intervals of -0.02 [-0.07, 0.04], 0.05 [0.02, 0.07], and 0.09 [0.07, 0.12] for the 1946, 1958, and 1970 birth cohorts, respectively). Increases in distress were larger among women than men, widening pre-existing sex inequalities (Difference-in-Differences and 95% confidence intervals of 0.17 [0.06, 0.28], 0.11 [0.07, 0.16], and 0.11 [0.05, 0.16] when comparing sex inequalities in the pre-pandemic peak in midlife to those observed by September/October 2020). As expected in cohort designs, our study suffered from high proportions of attrition with respect to the original samples. Although we used non-response weights to restore sample representativeness to the target populations (those born in the UK in 1946, 1958, and 1970, alive and residing in the UK), results may not be generalisable to other sections within the UK population (e.g., migrants and ethnic minority groups) and countries different than the UK.

Conclusions. Pre-existing long-term psychological distress trajectories of adults born between 1946 and 1970 were disrupted during the COVID-19 pandemic, particularly among women, who reached the highest levels ever recorded in up to 40 years of follow-up data. This may impact future trends of morbidity, disability, and mortality due to common mental health problems.

Comment 4.

Please include line numbering throughout your manuscript to facilitate future reviews.

Response 4.

We have included continuous line numbering throughout the manuscript.

Comment 5.

At this stage, we ask that you include a short, non-technical Author Summary of your research to make findings accessible to a wide audience that includes both scientists and non-scientists. The Author Summary should immediately follow the Abstract in your revised manuscript. This text is subject to editorial change and should be distinct from the scientific abstract.

Response 5.

As requested, we have included the Author Summary:

In text (lines 71-101):

- **Why Was This Study Done?**
  - The COVID-19 pandemic has negatively impacted the mental health of the population, with disproportionate effects among specific subgroups such as women and younger people.
  - Previous research suggests that, in the UK population, long-term trends of psychological distress are expected to reach their highest point during midlife (around age 30-45) and decrease towards older age.
  - Little is known about where the potential impact of the COVID-19 pandemic stands in relation to those long-term trends of psychological distress, and whether this impact has been different across cohorts and sexes.
- **What Did the Researchers Do and Find?**
  - We used data on 16,389 participants from three British birth cohorts representing people born in Britain in 1946, 1958, and 1970.
  - Using data on psychological distress collected as early as 1981 and up to February/March 2021, we measured the long-term psychological distress trajectories of different cohorts (people born in 1946, 1958, and 1970) and sexes (women and men).
  - We found that psychological distress levels increased during the COVID-19 pandemic, reaching or exceeding the highest levels ever recorded in up to 40 years of data, and that this increase was larger among women.
- **What Do These Findings Mean?**

- This study suggests that, during the COVID-19 pandemic, there has been a new peak in the long-term trajectories of psychological distress in the UK population, one that was largely unexpected considering pre-existing trends.
- This new peak in the psychological distress trajectories has been substantially larger in women than in men, widening the sex inequalities already existing prior to the pandemic onset.
- This new peak in distress may increase the trends of morbidity, disability, and mortality due to common mental health problems, with women likely being disproportionately affected. Public policies aimed at the provision of support and monitoring of population mental health, particularly among those most disproportionately affected by the pandemic, are needed to tackle existing and prevent future inequalities.

Comment 6.

Citations should be in square brackets, and preceding punctuation.

Response 6.

We have updated the citations accordingly.

Comment 7.

You may use almost any description as the item name of your supporting information as long as it contains an "S" and number. For example, “S1 Appendix” and “S2 Appendix,” “S1 Table” and “S2 Table,” and so forth (as opposed to ‘eAppendix #).

Response 7.

We have changed the description from eAppendix, eTable, and eFigure to Appendix S_, Table S_, and Figure S_ in the text and the Supplementary Material.

Comment 8.

Please specify whether informed consent was written or oral.

Response 8.

We have clarified in the text that oral informed consent was provided by the cohort members:

> ***In text (lines 159-160):*** All participants provided oral informed consent.

Comment 9.

Please remove the ‘Role of the funding source’ section.

Response 9.

We have removed this section from the text.

Comment 10.

Please ensure that the study is reported according to the STROBE guideline, and include the completed STROBE checklist as Supporting Information. Please add the following statement, or similar, to the Methods: "This study is reported as per the Strengthening the Reporting of Observational Studies in Epidemiology (STROBE) guideline (S1 Checklist)."

When completing the checklist, please use section and paragraph numbers, rather than page numbers.

Response 10.

Thank you for this suggestion. We have included the checklist as Checklist S1 in the Supplementary Material, and included the appropriate statement at the end of the Methods section:

> ***In text (lines 299-300):*** This study is reported as per the Strengthening the Reporting of Observational Studies in Epidemiology (STROBE) guideline (Checklist S1).

Comment 11.

Did your study have a prospective protocol or analysis plan? Please state this (either way) early in the Methods section.

Response 11.

Thank you for this comment. We have included the relevant statement at the end of the Data analyses section, as it refers to specific analytical strategies presented throughout this section:

In text (lines 294-298): Analyses were planned in May 2021. The use of projections for the psychological distress levels using data up to the most recent pre-pandemic assessment, alongside SMDs and DiD estimates, were included later on as a way of supplementing and summarising the evidence of the main analyses. Robustness checks using additional non-response weights across all data collection points were included as part of the revision process.

Comment 12.

Please define "lost to follow-up" as used in this study. Other reasons for exclusion should be defined.

Response 12.

We have clarified the reasons for exclusion in the Methods section:

In text (lines 148-150): Thus, participants lost to follow-up during the COVID-19 Survey (those who were no longer alive, not living in the UK, or not participating in any of the COVID-19 Survey waves) were excluded.

Comment 13.

Please define the length of follow up (eg, in mean, SD, and range), overall and by cohort.

Response 13.

We have included that information in the Results section:

In text (lines 308-313): Mean length of follow-up in the overall sample was 31.79 years (SD=8.88, with a minimum follow-up length of 0 years (as 63 participants only had information at one time point during the COVID-19 Surveys) and a maximum follow-up length of 40 years. Cohort-specific length of follow-up was M=37.64 (SD=5.01, range: 0-39) in NSHD; M=37.87 (SD=5.95, range: 0-40) in NCDS; and M=23.23 (SD=4.39, range: 0-25) in BCS70.

Comment 14.

Please include the date of access for references 43 and 46.

Response 14.

Thank you for this suggestion. We have clarified that the date included in the reference was the access date.

Comment 15.

Please remove the Declarations of interest, Author contributions and Data Availability section from the main text as this information is already captured as part of the submission form.

Similar to the above, please remove any funding information in your acknowledgements that is already covered in your Financial Disclosure (submission form).

Response 15.

We have removed the Declarations of interest, Author contributions, and Data availability sections from the main text.

Comment 16.

References: Journal name abbreviations should be those found in the National Center for Biotechnology Information (NCBI) databases.

Response 16.

We have updated the references in both the manuscript and the supplementary material as requested.

Comment 17.

Please use et al. after listing the first six authors in your Supporting Information references.

Response 17.

We have updated the references in the supplementary material as requested.

Reviewer 1.

Comment 1.

I would have liked to see a bit more on the policy implications of this work in the discussion, especially in the context of the UK.

Response 1.

Thank you for this comment. We have included a new paragraph in the Discussion focusing on the policy implications of our study in the UK:

> ***In text (lines 405-431):*** Overall, our study suggests that the COVID-19 pandemic had a major impact on the mental health of the UK adult population. The causal mechanisms driving those adverse effects are manifold, likely including the impact and fear of the disease and the lockdown measures and subsequent limitations to the usual day-to-day activities. However, the finding that some of the worst psychological distress levels observed during the pandemic did not take place during lockdown periods suggest, in line with previous evidence [8], that the lockdown measures are not the only –or even the main– factor driving those adverse effects. Rather, the larger-scale impacts of the pandemic on the people's and country's financial situation and on other disrupted systems such as health services (crucially including mental health services [45]), may be of great importance at explaining these adverse effects and why they remained or even worsened during non-lockdown periods. The results of our study partly align with evidence from countries such as the Netherlands where, almost a year after the pandemic onset, depressive and worry symptoms remained higher than before the pandemic onset in people with no record of psychiatric disorders, whereas anxiety symptomatology gradually returned to its initial levels [46]. However, the comparison of our findings with those from different countries (even those geopolitically similar to the UK) is difficult due to the overlap between the pandemic and the UK's exit from the European Union (Brexit) [47], as the role of these two in the UK population's mental health may be intertwined. The finding that women, already disadvantaged prior to the pandemic, experienced even worse effects points in the same direction, as such inequalities are unlikely to be solely due to the differential effects of the disease and the lockdown measures by themselves. Rather, as mentioned above, the widened sex inequalities likely reflect pre-existing differences in socialisation and oppression that may have been accentuated in pandemic times [39-42]. The results of our study highlight how public policies aimed at the provision of support and continued monitoring of

population mental health, particularly focused on the most disadvantaged groups (women, in our study), are very much needed to prevent further widening of inequalities. Furthermore, they serve as a warning for future lockdown-type measures to account for the differential impact of such measures in interaction with pre-existing oppression systems that may further jeopardise the mental health status of those most disadvantaged.

Reviewer 2.

Comment 1.

There is really only one minor change:

P4, Paragraph 2. "we aims to understand", should be "we aim to understand"

Response 1.

Thank you for noticing this typo. We have corrected it.

Comment 2.

eFig 3.1 Not a comment that needs a response - but I found the change in measure 7 very interesting. Public health is more than just health protection, and wellbeing is a vital part of health

Response 2.

Thank you for this comment. We totally agree with this and plan to conduct future studies focused on measures that capture (subjective) wellbeing rather than ill-being as the one used in this study (psychological distress).

Reviewer 3.

Comment 1.

While the authors depart from an interesting premise that mental distress caused COVID-19 pandemic should be viewed in the context of life course trajectories of mental distress, I am not totally convinced by the arguments provided:

Sentence: "they do not provide evidence on where these changes stand in relation to pre-existing long-term mental health trajectories" (p 4) is unclear. Changes in which direction? And what is meant by pre-existing trajectories? Can we assume that life course trajectories in previous generations form a template for how future generations will experience mental distress? Please clarify this.

Response 1.

Thank you for this comment. We have clarified the rationale and relevance of the study and what we mean by "pre-existing long-term mental health trajectories" in the text. By no means do we assume that life course trajectories in previous generations can be simply generalised to future generations, and this is precisely one of the reasons why we are exploring differences across generations (**lines 132-135**), and why we clarify throughout the text (e.g., **line 141** and **line 449**, and now also **lines 116-117**) that this study is focused on those cohorts born in the UK at particular years.

> ***In text (lines 110-130):*** Although these studies are crucial to understand whether population mental health has worsened during the pandemic, they do not provide evidence on where these changes stand in relation to pre-existing long-term mental health trajectories. In other words, how do psychological distress levels experienced during the pandemic compare to those experienced by the same individuals throughout their life course?
>
> The answer to this question is particularly important as psychological distress levels are expected to change with age. For instance, evidence prior to the pandemic using data from three British birth cohorts (those born in 1946, 1958, and 1970) has shown that, throughout adulthood, there seems to exist an upwards trend in the long-term psychological distress trajectories by middle age (age 30-45), and a decrease towards older age [14, 15]. Across these cohorts, the pandemic occurred at different life stages, with those born in 1970 experiencing or having recently experienced the midlife peak in distress, and those born in 1946 being further on in the decreasing trend towards older age. By extending the abovementioned life course analyses to include data collected during the first year after the COVID-19 pandemic onset, we aim to understand whether the changes in distress reflect a continuation or an alteration/disruption of these pre-pandemic trends (i.e., are the changes in line with the trends observed prior to the pandemic or not?), as well as provide relevant insights on the magnitude of the distress levels experienced during the pandemic by comparing them to the highest levels ever experienced. This may have important

implications for future trends of morbidity, disability, and mortality [2, 16], particularly in light of the most recent results of the Global Burden of Disease Study [2] which show that, right before the pandemic onset, common mental health problems remained among the leading causes of burden worldwide.

Comment 2.

It is unclear how the authors define a "disruption/alteration". Is a disruption defined a temporary elevation in psychological distress during the pandemic? If so, how does this add to the current literature? (as we already know that distress levels generally increases during the pandemic). Or is a disruption an elevation in distress symptoms compared to the highest distress levels measured in an individual's life course? If so what exactly does this tell us about how the life course trajectory of physical distress is altered because of the pandemic? (one suggestion to maybe make this more clear is to consider adding an graph displaying an hypothesized trajectory of the disruption in mental distress see for example Norris, F. H., Tracy, M., & Galea, S. (2009). Looking for resilience: Understanding the longitudinal trajectories of responses to stress. Social science & medicine, 68(12), 2190-2198.)

Response 2.

Thank you for this comment. We have clarified these points in response to the previous comment, showing how we are interested in situating the changes in distress with the pandemic onset (increasing levels, based on previous literature) in relation to both the levels experienced throughout the life course and the trends observed prior to the pandemic.

Comment 3.

It is unclear to me if the authors are interested in period, age or birth cohort differences throughout the manuscript. In part this is due to the fact that various terminology is used throughout. Please consider sticking with one term and formulate clearer expectations with regards to how disruptions are expected to differ depending in what life stage an individual experiences the COVID-19 pandemic (mid-life or after mid-life). Note if the aim is to investigate birth cohort differences, I would expect a broader analysis of how cohorts differ between each other beyond the age at which they experienced the pandemic (i.e. increased levels of education, socio-political changes, changed in sex inequalities across birth cohorts in general).

Response 3.

Thank you for this comment. Although we are interested in differences across cohorts (as well as across sexes), the aims of the study are eminently descriptive and, therefore, we do not refer to the differences across cohorts as “cohort effects”, as we agree that such interpretation (at least an interpretation that engages in the potential explanatory factors underlying those cohort differences) may require taking into account differences across cohorts beyond the age at the pandemic onset.

To avoid terminological confusion, we have removed the phrase “period effects” throughout the text (**lines 315**, **391**, and **462-464**). We have also clarified in the text that the pandemic occurred at different life stages across the studied cohorts:

> ***In text (lines 116-121):*** For instance, evidence prior to the pandemic using data from three British birth cohorts (those born in 1946, 1958, and 1970) has shown that, throughout adulthood, there seems to exist an upwards trend in the long-term psychological distress trajectories by middle age (age 30-45), and a decrease towards older age [14, 15]. Across these cohorts, the pandemic occurred at different life stages, with those born in 1970 experiencing or having recently experienced the midlife peak in distress, and those born in 1946 being further on in the decreasing trend towards older age.

Comment 4.

Some results/analysis that seem integral to answering the research question and aims are "hidden" in the appendix. This overall does not improve the readability of the paper. Moreover, it is not always clear to me where specific results can be found, which results are coming from which analysis and which results are connected to which aim. Overall, I have a few suggestions that might make the connection between the appendix and the text more clear.

eAppendix 1 is mentioned in the "sample and procedures" section on page 5. However, it is not mentioned what the content of this appendix is and how it adds to the description given on this page.

Response 4.

Thank you for this comment. We have removed the reference to this appendix in the Sample and procedures subsection, and kept it in the Measures subsection, where its content and what it adds to the text was already detailed.

Comment 5.

Consider reporting eAppendix 6 in the methods section as this is an integral part of the research aim.

Consider reporting eAppendix 7 in the sample description part of the methods section.

Consider reporting eAppendix 8 in the methods section.

Response 5.

Thank you for these suggestions. We have now included the three appendices in the main text. Former eAppendix 6 is now included in the Data analyses subsection; former eAppendix 7 is now Figure 1, referenced in the Results section; and former eTable 8.1 is now Table 2, referenced in the Results section as well. To keep consistency, we have also included in the main text information on the additional psychological distress operationalisations from the eAppendix S3, relevant to understand the text moved from former eAppendix 6 to the Data analyses subsection.

Comment 6.

I think it would greatly enhance readability if conclusions of the analysis found within the appendix are mentioned in the text and not just where you can find the information. For example, on page 6 you mention that further details on the measurement invariance testing procedure can be found in eAppendix 4. However you do not mention whether various psychological distress levels were invariant across cohorts. This creates an unnecessary cliff-hanger. Please consider mentioning the conclusions of appendices in the text.

Response 6.

Thank you for this comment. We have clarified under the subheading on Measurement invariance/equivalence testing the main results from these analyses:

> ***In text (lines 209-211):*** Evidence on measurement invariance up to the required level to perform the subsequent analyses (i.e., scalar invariance) was obtained, and further details on the procedure used, along with its results, are available in Appendix S4.

Comment 7.

Overall the method section could use more structure in terms of which analysis/results section focuses on which aim. The same holds true for the result section. For example, in section titled "trajectories of psychological distress" it is not clear why the analysis discussed at the end of the page (SMD) was done and which research aim it was supposed to answer to. In addition it was unclear how the two relevant pre-pandemic time-points were determined. Another example, is in the beginning of the result section where it is not clear where I do not find it clear the result "A period effect was observed …across the cohorts" (p8) can be found and which aim it related to. Please clarify this.

Response 7.

Thank you for this comment. In the Methods section, we have included the specific aim of the analyses at the start of the subsection “Trajectories of psychological distress”, and included a new subsection entitled “Comparison of distress levels during the pandemic with most recent and highest levels”, also starting with the specific aim of the analyses:

> ***In text (lines 224-228):*** To understand whether the changes in distress reflect a continuation or an alteration/disruption of the pre-pandemic trends under the different outcome operationalisations, we used a multilevel growth curve modelling approach, using linear models for the factor scores operationalisations (continuous), Poisson models for the number of symptoms operationalisation (discrete), and logistic models for the ‘caseness’ operationalisation (binary).
>
> ***In text (lines 261-263):*** To address the question of how the levels of distress experienced during the pandemic compared to both recent pre-pandemic levels and also to the highest levels recorded in the cohort-specific trajectory, we obtained the standardised mean differences (SMD)…

In the Results section, we have removed the reference to the “period effect” (as per Response 3), and included a new subsection entitled “Comparison between levels during the pandemic and pre-pandemic levels”.

Comment 8.

While the results cover a representative population in the UK, it would be interesting if you could reflect on the meaning of these results for other countries that have experienced lockdowns. For example, in studies conducted in the Netherlands (e.g. Kok AA, Pan K-Y, Ottenheim NR, Jörg F, Eikelenboom M, Horsfall M, et al. Mental health and perceived impact during the first Covid-19 pandemic year: A longitudinal study in

Dutch case-control cohorts of persons with and without depressive, anxiety, and obsessive-compulsive disorders. Journal of affective disorders. 2022) showed that mental distress levels gradually return to its initial levels, especially with regards to depression and anxiety. Does this mean that the UK potentially stands out in this regard? If so why?

Response 8.

Thank you for this suggestion. Due to differences in the sampling strategies and target populations, it is difficult to compare the results of our study with other studies conducted within and outside of the UK. Furthermore, the overlap between the pandemic and the UK's exit from the European Union make the comparison with non-UK countries even harder, as the effects of these two may be intertwined. We have, however, compared our results with those from the suggested reference, and highlighted the limitations to the comparisons with this and other studies, in the Discussion section:

> ***In text (lines 414-421):*** The results of our study partly align with evidence from countries such as the Netherlands where, almost a year after the pandemic onset, depressive and worry symptoms remained higher than before the pandemic onset in people with no record of psychiatric disorders, whereas anxiety levels were similar [46]. However, the comparison of our findings with those from different countries (even those geopolitically similar to the UK) may be difficult due to the overlap between the pandemic and the UK's exit from the European Union (Brexit) [47], as the role of these two in the UK population's mental health may be intertwined.

Comment 9.

Abstract:

Second sentence: There is an illogical comparison. "distress typically rises until mid-life and then falls [after mid-life] in both sexes." Please add "after mid-life"

Response 9.

Thank you for this suggestion. We have changed the text accordingly:

> ***In text (lines 35-37):*** The extent that these changes have altered common age-related trends in psychological distress, where distress typically rises until midlife and then falls after midlife in both sexes, is unknown.

Comment 10.

Abstract:

Methods: please mention the procedures/analytic method in the abstract

Response 10.

We have included that information in the Abstract:

> ***In text (lines 47-53):*** We used a multilevel growth curve modelling approach to model the trajectories of distress across cohorts and sexes and obtained estimates of the differences between the distress levels observed during the pandemic and those observed at the most recent pre-pandemic assessment and at the peak in the cohort-specific pre-pandemic distress trajectory, located at midlife. We further analysed whether pre-existing cohort and sex inequalities had changed with the pandemic onset using a Difference-in-Differences approach.

Comment 11.

Introduction:

"we aims to understand (..)" (p 4) . The s after aim should be removed.

Response 11.

Thank you for noticing this typo. We have corrected it.

Comment 12.

Methods:

How was the survey administered - online?

What was the response rate?

Response 12.

We have included this information in the Sample and procedure subsection:

> ***In text (150-155):*** Data collection for the COVID-19 Survey was entirely online at the first and second time-points and was supplemented by telephone interviews at the third time-point. Response rates to the COVID-19 Survey with respect to the target population (cohort members alive and still residing in the UK) in NSHD, NCDS, and BCS70 were 31.1%, 33.5%, and 23.6% in the first wave; 39.6%,

40.7%, and 29.9% in the second wave; and 35.3%, 44.2%, and 32.5% in the third wave, respectively [21].

Comment 13.

Methods:

Please consider "choice of primary measure" changing into "mental distress measure"

Response 13.

We have removed this subheading as the current heading Measures already captures the content of the paragraph.

Comment 14.

Methods:

Please explain which "outcome operationalisations" are used in the multilevel growth curve modelling in page 7.

Response 14.

Thank you for this suggestion. We have clarified that the main analyses are conducted using the cross-cohort factor score operationalisation, and that additional operationalisations were used as sensitivity checks:

> ***In text (lines 178-180):*** Due to the wide range of different measures of psychological distress across cohorts (NSHD vs NCDS and BCS70) and within NSHD, we operationalised psychological distress as a factor score (continuous).
>
> ***In text (lines 186-196):*** As sensitivity checks, we used additional psychological distress operationalisations, in addition to the main operationalisation as a factor score. First, we operationalised psychological distress as the number of symptoms present (discrete) at each time-point. This could be directly done in NCDS and BCS70 due to the use of the same instrument across cohorts and over time; and relied on three out of the seven previously harmonised symptoms that were present across all data collection points in NSHD due to the change in the version of the GHQ used in the COVID-19 Survey. Thus, the potential number of symptoms ranged from 0 to 9 in NCDS and BCS70, and from 0 to 3 in NSHD. Second, psychological distress was operationalised as 'caseness' (binary), using each of the measurement tools' recommended thresholds (Appendix S1). Finally,

an additional factor approach was implemented in NSHD using the seven previously harmonised symptoms as indicators of a latent psychological distress factor. Further details on these additional psychological distress operationalisations are available in Appendix S3.

Comment 15.

Methods:

How many missings where there in the pre-COVID data and how were they handled?

Response 15.

Thank you for this comment. We have included below a table with the number of missing observations at each wave, conditional on meeting the inclusion criteria at the time of the study (i.e., being alive and still residing in the UK, and having participated in at least one of the COVID-19 Survey waves). Our analyses depart from the assumption that missingness in the pre-pandemic data is random conditional on meeting those inclusion criteria at the time of the study. This may be particularly surprising when looking at the number of missing observations in the January/March 2020 sweep in NCDS, but since missingness in this sweep was driven by the cancellation of data collection due to the pandemic onset, which was unrelated to the study participants' characteristics, we are confident the assumption holds even in this case.

	Age	Year	Number of missing observations
NSHD	36	1982	211
	43	1989	133
	53	1999	171
	63	2009	366
	69	2015	316
	74	May 2020	409
	74·5	Sept/Oct 2020	167
	75	Feb/Mar 2021	357
	**Age**	**Year**	**Number of missing observations**
NCDS	23	1981	1059
	33	1991	931
	42	2000	583
	50	2008	756
	61·7	Jan/Mar 2020	6196
	62	May 2020	2647
	62·5	Sept/Oct 2020	1362

	63	Feb/Mar 2021	867
	**Age**	**Year**	**Number of missing observations**
**BCS70**	26	1996	1678
	29	1999	867
	34	2004	1200
	42	2012	1164
	46	2016	1183
	50	May 2020	2865
	50·5	Sept/Oct 2020	1777
	51	Feb/Mar 2021	1326

However, to challenge this assumption, we have extended the derivation of the non-response weights to the pre-pandemic data collection points. In order to do so, and in line with the procedure used for the derivation of the analogous non-response weights in the COVID-19 Surveys (available in the COVID-19 Survey User Guide already referenced in the main text), we have used early life covariates (birth sex, housing tenure and crowding, parental social class during childhood, and cognitive ability), along with the number of non-responses to previous data collection points, to predict the probability of non-response. The resulting probabilities have been used as inverse probability weights (IPW) in the main analyses (multilevel growth curve models using the cross-cohort psychological distress factor operationalisation). The results of these analyses are similar to those already reported in the manuscript.

We have detailed the rationale for these additional analyses in the Methods section, reported the table above (number of missing observations by wave and cohort) in the Supplementary Material (Appendix S5), and briefly commented on the robustness of the results in the Results section, including the detailed results in the Supplementary Material (Appendix S7):

> ***In text (lines 280-290):*** Missingness in pre-pandemic data collection points was assumed to be random conditional on meeting the inclusion criteria (i.e., being alive and still residing in the UK, and having participated in at least one of the COVID-19 Survey waves) at the time of the study. However, as a robustness check, we derived non-response weights for the pre-pandemic data collection points following a similar procedure as the one laid out in the COVID-19 Survey User Guide [21]. We used information on early life variables (birth sex, housing tenure and crowding, parental social class during childhood, and cognitive ability), along with the number of non-responses to previous data collection points, to predict the probability of non-response to the pre-pandemic data collection points. The resulting probabilities were used in an IPW approach to

estimate the multilevel growth curve models using the main psychological distress operationalisation (cross-cohort factor score), and the results were compared to those of the main analyses.

In text (lines 352-356): Analyses performed using the cross-cohort factor score operationalisation including non-response weights at all time-points (Appendix S7), and those with the observed 'number of symptoms' operationalisation (Appendix S8), the 'caseness' operationalisation (Appendix S9), and the factor scores derived from the seven harmonised indicators within NSHD (Appendix S10) provided very similar results as those found in the main analyses.

Comment 16.

Results

Consider adding a Table depicting descriptive statistics in the beginning of the result section. (particularly would it be possible to show whether the three samples differed with regards to other background characteristics: educational level, income, overall health)

Response 16.

Thank you for this suggestion. We have included that information in the manuscript (Table 1), along with an overview of the measures used in the Methods section and a short comment on the descriptive results in the Results section:

In text (lines 198-202): Information on the highest vocational/academic qualification level achieved (harmonised into National Vocational Qualification [NVQ] levels according to the procedure laid out in Dodgeon and Parsons [33]), along with the self-reported financial situation before the COVID-19 pandemic and the self-reported general health level (both collected during the COVID-19 Survey waves), was used to provide descriptive information on the samples.

In text (lines 305-307): Members of younger cohorts had higher vocational/academic qualification levels and reported better general health levels and worse pre-pandemic financial situation than members of older cohorts (Table 1).

Comment 17.

Overall:

The variable use of birth cohort, age, generation and period difference is confusing. Consider sticking with the term birth-cohort instead of generation throughout the paper.

Response 17.

Thank you for this comment. In addition to the changes reported in Response 3, we have replaced the word “generation” with “cohort” throughout the text.

Reviewer 4.

Comment 1.

Page 6 - "The item harmonisation procedure reported elsewhere(14, 29) was implemented where items from these different questionnaires were mapped to specific distressing experiences." I would like the authors to make this a bit clearer here about what was done without relying on citations. Even just a sentence summary would suffice. What do the authors mean exactly by 'mapped to specific distressing experiences'? Do they mean symptoms? eAppendix 2 makes sense but nothing here mentioned about the process.

Response 1.

Thank you for this comment. We have clarified in the text what was done as part of the mapping of items to specific distressing experiences:

> ***In text (lines 171-174):*** The same item harmonisation procedure implemented by McElroy et al. [29] was used. Following this procedure, items from the GHQ-12 questionnaire, administered during the COVID-19 Survey, were mapped to specific distressing experiences, including low mood, fatigue, tension, panic, hopelessness, health anxiety, and sleep problems.

Comment 2.

Page 6 - "Information on the cohort members' biological sex as recorded at birth was used." used for what? Used how?

Response 2.

We have further clarified this in the text:

> ***In text (lines 197-198):*** Information on the cohort members’ biological sex as recorded at birth was used in the interaction analyses by birth sex.

Comment 3.

eAppendix 5 - this SEM diagram does not really make sense to me. What are the dotted boxes vs the solid boxes? Are they latent constructs of the scales? How was an SEM model run to associate or path one to another when usually measure-type completed is mutually exclusive by survey? I have never seen an SEM model illustrated like this.

Response 3.

Thank you for this comment. We have removed the diagram from the supplementary material, as the diagram may not be as helpful as we intended and the procedure we were trying to visually represent is already laid out in the main text (**lines 178-185**).

Comment 4.

I appreciate the authors used non-response weights but did the authors investigate whether there was any non-response bias during the COVID surveys? Any significant pre-pandemic differences by demographics or prior psychological distress?

Response 4.

There were differences across subgroups in the likelihood of them participating in the COVID-19 Surveys. Among them, women and cohort members with higher educational/vocational qualification levels were more likely to participate than men and cohort members with lower educational/vocational qualification levels or no qualifications, whereas no significant differences were found by pre-pandemic psychological distress. We have included this information in the text, indicating that further details on these differences are available in the COVID-19 Survey User Guide:

> ***In text (lines 269-273):*** There were differences within the cohorts in the probability of participating in the COVID-19 Survey waves. Women and cohort members with higher educational/vocational qualification levels were more likely to participate in the survey than men and members with lower qualification levels or no qualifications, but no significant differences were found by pre-pandemic psychological distress (more details are available in Appendix 1 of the COVID-19 Survey User Guide [21]).

We have also further clarified that the weights derived to address this differential probability of participating are effective to restore sample representativeness and to reduce bias:

In text (lines 278-280): Additional information on the derivation of these weights and their effectiveness to restore sample representativeness and reduce bias is available in the COVID-19 Survey User Guide [21].

We are thankful for the interest shown in our study, and for the very insightful comments. We believe that, thanks to these comments and the revisions we have made to address them, the manuscript is now stronger.

We look forward to hearing from you.

Sincerely,

Darío Moreno-Agostino, PhD
Centre for Longitudinal Studies, Social Research Institute, University College London, UK
ESRC Centre for Society and Mental Health, King's College London, UK
d.moreno@ucl.ac.uk
