# Supplementary material for: Long-term psychological distress trajectories and the COVID-19 pandemic in three British birth cohorts: A multi-cohort study
Source: PLoS Med. 2023 Apr 4;20(4):e1004145. doi: 10.1371/journal.pmed.1004145 (PMC10072377; doi:10.1371/journal.pmed.1004145)
Supplement: Attachment — Submitted filename: Response to reviewers.pdf [file pmed.1004145.s003.pdf]

November 8, 2022

Prof Vikram Patel, Prof Toshi A Furukawa,
Prof Lola Kola, and Prof Daisy Fancourt

Guest Editors, *PLOS Medicine*

Dear Professors Patel, Furukawa, Kola, and Fancourt,

We appreciate the further comments made by the editors and reviewers on the manuscript “Long-term psychological distress trajectories and the COVID-19 pandemic in three British birth cohorts: a multi-cohort study” (reference: PMEDICINE-D-22-01955R2), submitted to the *PLOS Medicine* call for papers on *COVID-19 Pandemic and Global Mental Health*.

We were glad that the revisions made to the manuscript were found appropriate and thank the editors and reviewers again for their time and further suggestions. Please find our responses to these below:

Editors.

Comment 1.

Please ensure that all numbers presented in the abstract are present and identical to numbers presented in the main manuscript text.

Response 1.

We have double-checked and confirmed this. However, to clarify which specific estimates are being provided, we have included in the abstract the ‘labels’ with which they are referred to in the main manuscript text:

> ***In text (lines 53-61):*** By September/October 2020, distress levels had reached or exceeded the levels of the peak in the pre-pandemic life-course trajectories, with larger increases in younger cohorts (Standardised Mean Differences [SMD] and 95% confidence intervals of $SMD_{NSHD,pre\text{-}peak}$=-0.02 [-0.07, 0.04], $SMD_{NCDS,pre\text{-}peak}$=0.05 [0.02, 0.07], and $SMD_{BCS70,pre\text{-}peak}$=0.09 [0.07, 0.12] for the 1946, 1958, and 1970 birth cohorts, respectively). Increases in distress were larger among women than men, widening pre-existing sex inequalities (Difference-in-

Differences [DiD] and 95% confidence intervals of $DiD_{NSHD,sex,pre\text{-}peak}$=0.17 [0.06, 0.28], $DiD_{NCDS,sex,pre\text{-}peak}$=0.11 [0.07, 0.16], and $DiD_{BCS70,sex,pre\text{-}peak}$=0.11 [0.05, 0.16] when comparing sex inequalities in the pre-pandemic peak in midlife to those observed by September/October 2020).

Comment 2.

Lines 418-421: I would propose that this addition requires some elaboration and should be supported by references if possible.

Response 2.

Thank you for this suggestion. This addition was intended to build up on the previous lines referring to the larger-scale impacts of the pandemic on the financial situation and health services performance. We have clarified this in the text by specifying that UK's exit from the European Union may have had an impact on some of the same systems, including a supporting reference:

> ***In text (lines 422-428):*** However, the comparison of our findings with those from different countries (even those geopolitically similar to the UK) may be difficult due to the overlap between the pandemic –with the first wave of COVID-19 and introduction of restrictions happening in March 2020 [47]– and the UK's exit from the European Union (Brexit) –with the transition period taking place for most of 2020 and the UK leaving the European Union on the 1st of January 2021 [48]. The role of these two events on the abovementioned financial and health services systems may be intertwined and difficult to disaggregate as they both have been happening roughly at the same time [49].

Reviewer 3.

Comment 1.

Author summary: Under the heading "what did the researchers do and find" the statement "as early as 1981" seems rather unclear. Given the emphasis of changes over de life course especially during midlife, I think it may be important to mention the age ranges and cohort memberships (in terms of birth year) of respondents included in the study in the author summary. Please consider adding this information.

Response 1.

Thank you for this suggestion. We have included that information in the Author Summary:

In text (lines 84-87):

- We used data on 16,389 participants from three British birth cohorts representing people born in Britain in 1946, 1958, and 1970, with data on psychological distress collected between 1982-2021 (age 36-75), 1981-2021 (age 23-63), and 1996-2021 (age 26-51), respectively.

Comment 2.

Author summary: In the "what do the findings mean?" section authors may consider more clearly stating that the word "new" before peak refers to the fact that this peak was found in addition to the peak already observed in midlife (e.g.: additional peak, second peak, in addition to the peak in midlife, we now also find…).

Response 2.

Thank you for this suggestion. We have clarified this in the Author Summary:

In text (lines 94-97):

- This study suggests that, during the COVID-19 pandemic, there has been a new peak in the long-term trajectories of psychological distress in the UK population, one that was largely unexpected considering pre-existing trends, in addition to the peak already observed in midlife.

Comment 3.

Methods: It is not clear to me how old participants were at the first measurement included in the study (i.e. various figures suggest NHD: 35, NCDS: between 25-30, BCS: 20-25). Please consider adding baseline age for each cohort to Table 1

Response 3.

Thank you for this suggestion. We have clarified this in the Author Summary (as per the response to Comment #1), and included this information in the main text:

In text (lines 166-167): We used data on psychological distress collected between 1982-2021 (NSHD, age 36-75), 1981-2021 (NCDS, age 23-63), and 1996-2021 (BCS70, age 26-51).

We have also included the baseline age of the modelled trajectories in the note of Table 2:

In text (Table 2, Note): The intercept corresponds to the age at the first collection of psychological distress data in adulthood, being age 36 in NSHD, age 23 in NCDS, and age 26 in BCS70.

Comment 4.

Methods: It is not clear to me what "age" means in table S5, Is this the average age of respondents who dropped out? What would be more informative perhaps is the average age of respondents who dropped out of the study compared to the average age of respondents included in the study.

Response 4.

Thank you for this comment. The table in Appendix S5 includes the number of missing observations by cohort at each wave. Since these are birth cohorts including data from participants born in the same week, the age of participants is homogeneous across waves within cohorts. We have included this clarification in the table note:

In text (Appendix S5, Supplementary Material, Note): Since each of the cohorts include data from participants born in the same week, the participants' age is homogeneous across waves/calendar years within cohorts.

Comment 5.

Overall: In response to comment 3 by reviewer 3 the authors mention that their paper aimed to "explore and describe" differences between cohorts. However, this is not reflected in the wording of the aim where the authors refer to "investigate" difference. Please consider using wording such as "explore/describe" in order to stay closer to the what was mentioned in the response.

Response 5.

Thank you for this suggestion. We have changed the wording accordingly:

In text (lines 136-138): By analysing these long-term psychological distress trajectories across cohorts and sexes, we also aim to explore whether there are inequalities in the potential disruption of the pre-existing long-term trends across cohorts and sexes.

Reviewer 4.

Comment 1.

My only additional query was whether the authors had explored whether there was a sex X pre-pandemic psychological distress interaction on missingness. Even if one is found it would not be critical, just interesting to report. Non-response seems to becoming a larger issue with time and so the more information on this in the public domain the better.

Response 1.

Thank you for this comment. We did not explore whether there was a sex * pre-pandemic psychological distress interaction on missingness. The variable selection for the assessment of missingness was based on the Centre for Longitudinal Studies' Missing Data Strategy described in Mostafa et al. (2021), which is data-driven and aimed at maximising the plausibility of the missing-at-random assumption. We agree that information on variables linked to non-response is of great relevance, and this is why this information is provided in great detail for these and additional cohort studies in the COVID-19 Survey User Guide (Brown et al., 2021) referenced in our manuscript.

- Mostafa et al., 2021: https://doi.org/10.1016/j.jclinepi.2021.02.019
- Brown at al., 2021: https://doc.ukdataservice.ac.uk/doc/8658/mrdoc/pdf/ucl_cohorts_covid-19_survey_user_guide.pdf

We are thankful for the time the editors and reviewers have taken to check our manuscript again, and for the additional helpful comments.

We look forward to hearing from you.

Sincerely,

Darío Moreno-Agostino, PhD

Centre for Longitudinal Studies, Social Research Institute, University College London, UK

ESRC Centre for Society and Mental Health, King's College London, UK

d.moreno@ucl.ac.uk
